# Comprehensive chemo-profiling of coumarins enriched extract derived from *Aegle marmelos* (L.) Correa fruit pulp, as an anti-diabetic and anti-inflammatory agent

**DOI:** 10.1016/j.jsps.2023.101708

**Published:** 2023-07-25

**Authors:** Ritu Tiwari, Smita Mishra, Gnanabhaskar Danaboina, Gaurav Pratap Singh Jadaun, M. Kalaivani, Vivekanandan Kalaiselvan, Mahaveer Dhobi, Rajeev S Raghuvanshi

**Affiliations:** a Indian Pharmacopoeia Commission, Ministry of Health & Family Welfare, Government of India, Raj Nagar, Ghaziabad 201002, India; b Department of Pharmacognosy and Phytochemistry, Delhi Pharmaceutical Sciences and Research University, New Delhi 110017, India; c Drugs Controller General of India, Central Drugs Standard Control Organization, FDA Bhawan, Kotla Road, New Delhi 110002, India

**Keywords:** *Aegle marmelos*, Bioactive markers enrichment, Coumarins, qNMR, ADMET, PPI

## Abstract

*Aegle marmelos* (L.) Correa is an Indian medicinal plant known for its vast therapeutic activities. In Ayurveda, the plant is known to balance “*vata*,” “*pitta*,” and “*kapha*” dosh. Recent studies suggest anti-inflammatory, anti-microbial, and anti-diabetic potential but lack in defining the dosage over the therapeutic activities. This study aims to determine the chemical profile of *Aegle marmelos* fruit extract; identification, enrichment, and characterization of the principal active component(s) having anti-inflammatory and anti-diabetic potential. Targeted enrichment of total coumarins, focusing on marmelosin, marmesin, aegeline, psoralen, scopoletin, and umbelliferone, was done from *Aegle marmelos* fruit pulp, and characterized using advanced high-throughput techniques. *In vitro* and *in silico* anti-diabetic and anti-inflammatory activities were assessed to confirm their efficacy and affinity as anti-diabetic and anti-inflammatory agents. The target compounds were also analysed for toxicity by *in silico* ADMET study and *in vitro* MTT assay on THP-1 and A549 cell lines. The coumarins enrichment process designed, was found specific for coumarins isolation as it resulted into 48.61% of total coumarins enrichment, which includes 31.2% marmelosin, 8.9% marmesin, 4% psoralen, 2% scopoletin, 1.7% umbelliferone, and 0.72% aegeline. The quantification with HPTLC and qNMR was found to be correlated with the HPLC assay results. The present study validates the potential use of *Aegle marmelos* as an anti-inflammatory and anti-diabetic agent. Coumarins enriched from the plant fruit have good therapeutic activity and can be used for Phytopharmaceutical ingredient development. The study is novel, in which coumarins were enriched and characterized by a simple and sophisticated methodology.

## Graphical abstract

**Figure:**
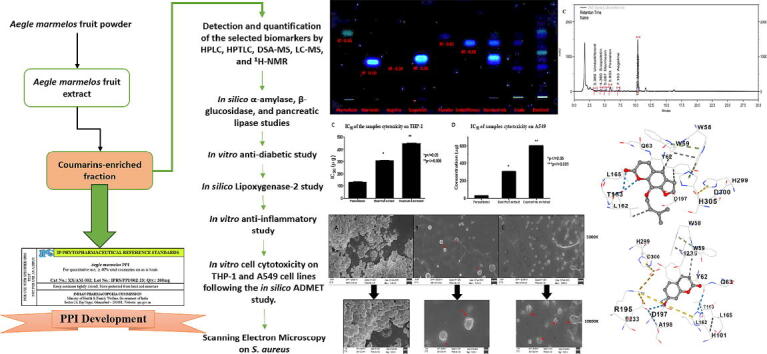

## 1 Introduction

Ayurveda is an ancient Indian medicinal system which has enlisted and described hundreds of diseases and their cure from medicinal plants. (Chauhan et al., 2015, Kumar et al., 2017, Sri Kirshan Dass Ji, 1939). One of these medicinal plants, *Aegle marmelos* (L.) Correa (Family: Rutaceae) is known for its various therapeutic activities, as Rig Veda and Charak Samhita described. *Aegle marmelos*, commonly known as Bael, Bengal quince or Golden apple, is indigenous to the Indian sub-continent and has spiritual and religious significance in Hinduism (Dass, 1939). In Ayurveda, the unripe fruit is known to balance *kapha* and *vata* dosh (Bhardwaj and Nandal, 2015, Sri Kirshan Dass Ji, 1939). In comparison, the young leaves (Bilva patra) are known to balance all three doshas and relieve pain, dyspepsia, gastritis, and abdominal colic pain (Sarkar et al., 2020). Its roots improve digestion, while the stem is efficacious for rheumatoid arthritis and heart disease (Shastri and Chaturvedi, 2004, Sri Kirshan Dass Ji, 1939). *Aegle marmelos* fruit powder is known to be one of the necessary items in Ayurvedic formulations. Protective effects against the wound, radiation, microbes, free radical generation, and depression have also been exhibited by *Aegle marmelos*. These records prove the natural healing power of *Aegle marmelos*. (Bhardwaj and Nandal, 2015, Sarkar et al., 2020).

Recent research and clinical studies on the crude extracts of the various plant parts showed the anti-diarrhoeal, anti-microbial, antiviral, anticancer, chemopreventive, antipyretic, analgesic, anti-ulcerative, diuretic, antifertility and anti-inflammatory properties (Choudhary et al., 2021). *Aegle marmelos* fruit has been reported to contain some valuable phytoconstituents such as marmelosin, marmelide, psoralen, alloimperatorin, rutaretin, scopoletin, aegeline, umbelliferone, marmelin, fagarine, anhydromarmelin, limonene, α-phellandrene, betulinic acid, marmesin, luvangentin and auroptene. A study has reported that the fruit pulp of the plant contains a high quantity of tannins and riboflavins also (Bheeman et al., 2014, Rahman and Parvin, 2014, Reddy and Urooj, 2013, Tiwari et al., 2022).

Despite the popularity of this plant, a systematic phytocompound(s) enrichment, chemical characterization, and bioactivity assessment have not been reported simultaneously in the scientific literature. The present study aims to enrich the principal active compounds in *Aegle marmelos* (L.) Correa fruit pulp, targets anti-inflammatory and anti-diabetic activity, leading to the development of a Phytopharmaceutical ingredient (PPI) with a defined dosage based on *in silico* and *in vitro* assays. The extracts were characterized and quantified by HPTLC, HPLC, DSA-MS, LC-MS, and qNMR. This study developed the process regarding crude extract enrichment, which can further be implemented for the isolation of compound(s) of interest. By using the advanced *in silico* approach, the target analytes were evaluated for their drug-likeness. *In vitro,* cytotoxic assay and Scanning Electron Microscopy (SEM) analysis confirm the *in silico* results. The study is holistic and can be implemented further for drug development and design.

## 2 Materials and methods

### 2.1 Plant material collection and authentication

For the present study, dried fruits of *Aegle marmelos* was bought from the local market and authenticated from CSIR-NIScPR (authentication no. – NISCAIR/RHMD/Consult/2021/4160–61-1). The fruits was coarsely powdered and stored in an airtight container for further use.

### 2.2 Chemicals and reagents

Solvents used for experimentation, such as ethanol, methanol, ethyl acetate, Dimethylsulfoxide (DMSO) and chloroform, were of analytical grade, purchased from Finar Ltd. and Merck. HPLC and LC-MS grade solvents were purchased from Merck. Marmelosin, marmesin, aegeline, psoralen, scopoletin, and umbelliferone were purchased from Sigma Aldrich. 3-(4,5-dimethylthiazol-2-yl)-2,5-diphenyltetrazolium bromide (MTT), trypsin, Bovine Serum Albumin (BSA), Glutaraldehyde solution for SEM analysis, Ammonium Molybdate, and Sodium Phosphate were purchased from SRL Chemicals. Ascorbic acid, α-amylase enzyme and trypsin were purchased from Finar and SRL chemicals. Deuterated solvent DMSO-D6 was purchased from Sigma Aldrich.

### 2.3 Extraction and coumarins enrichment

The pharmacognostic tests (loss on drying, total ash, acid-insoluble ash, and water & ethanol -soluble extractive values) of the fruit powder of *Aegle marmelos* were assessed and then about 5.0 g was accurately weighed and extracted with 50 mL methanol by refluxing at 65 °C for 2 h, cooled and filtered. The residue was extracted three times with the same procedure to get maximum yield. The filtrate obtained was combined and concentrated to dryness using a rotary vacuum evaporator, dried in a vacuum oven and extract yield was calculated on the dried basis.

The coumarins enrichment was done by following a method described for marmelosin isolation. After some modifications and many trials, the enrichment process for coumarins was designed (Shinde and Laddha, 2012). A portion of the dried extract was kept for screening studies; the remaining was dissolved in 10% w/v NaCl solution in water and filtered. First, the filtrate was partitioned with ethyl acetate repeatedly, and an organic layer was collected. Subsequently, the aqueous layer was acidified with acetic acid to pH-4 and again partitioned with ethyl acetate, followed by aqueous layer basification with ammonia solution to pH-9 and partitioned with ethyl acetate. Finally, all three organic layers were combined and concentrated to obtain coumarins enriched extract. Further purification was done using Vacuum Liquid Chromatography (VLC), as explained in Supplementary Figure 1.

### 2.4 *In vitro* anti-hyperglycaemic activity

To determine the anti-diabetic potential of the crude and enriched extracts, α-amylase Inhibition Assay was performed. Briefly, the test samples (in series of 100 to 1000 µg/ml) were added to the α-amylase enzyme and incubated for 10 min at 37 °C. Afterwards, the starch solution (1% w/v), i.e., the substrate was added to the mixture and incubated for 10 min. Finally, the DNS reagent was added to the solution and incubated for 10 min at 37 °C. The reagent colour changes to orange/deep red in the presence of sugar formation, which was measured by UV–Visible spectrophotometer at 540 nm (Ali et al., 2006, Mishra et al., 2019, Tamil et al., 2010).

### 2.5 *In vitro* anti-inflammatory activity

Test samples were checked for protease inhibitory response by pre-incubating 1 mL Trypsin (10 U/mL) with 1 mL of a series of samples (100 µg/ml to 1000 µg/ml) at 37° C for 15 min. 2 mL of 1% Bovine Serum Albumin (BSA) (0.1 M phosphate buffer) was added and incubated at 37° C for 30 min. The reaction was terminated by adding 2.5 mL of 0.44 M Trichloroacetic acid (TCA). The reaction mixture was centrifuged at 10,000 rpm for 15 min, and the pellet was discarded. The absorbance of the supernatant was measured at 280 nm (Hashmi et al., 2022, Leelaprakash and Mohan Dass, 2011, Sakat et al., 2010).

### 2.6 *In silico* studies

For the docking study, the proteins were prepared by Autodock tools, protein docking was done using CBdock, and the protein-drug interaction was visualized by Biovia Studio. The *in silico* anti-diabetic study was assessed on three proteins viz., α-amylase (2QV4), β-glucosidase (2ZOX), and pancreatic lipase (2OXE). The targeted molecules viz., marmelosin, marmesin, aegeline, psoralen, scopoletin, and umbelliferone in *Aegle marmelos* fruit pulp were docked with the proteins as mentioned earlier along with the standard drugs suitable as the enzyme inhibitors **(**Supplementary Figure 2). For α-amylase, acarbose was selected; for β-glucosidase – miglitol, and pancreatic lipase – sibutramine was selected. To determine the intestinal absorption, drug-likeness, volume distribution, metabolism, renal clearance, and potential hepatotoxicity, the ADMET test was performed using PkCSM software. The molecules were also evaluated for the Lipinski rule of 5 violations by the software provided by IIT-Delhi “SCFBio.” OSIRIS tool was used to evaluate the drug score and Molinspiration for bioactivity factor.

### 2.7 *In vitro* cell cytotoxicity assay

The cytotoxicity profiling of the crude and enriched extract of *Aegle marmelos* fruit powder was checked on THP-1 (human monocytic cell lines) and A549 (adenocarcinomic human alveolar basal epithelial cells) cell lines. Cell lines were maintained and cultivated in sterile Biosafety lab-2 (BSL-2) in a CO_2_ incubator. For THP-1 and A549 cell lines, RPMI1640 and DMEM media with 10% Foetal Bovine Serum (FBS) with 1% Pen-strep solution (Penicillin-streptomycin solution) was used at 37 °C and 5% CO_2_. For the MTT assay, cells were transferred in 96 well plates (5000–7000 cells per well) and incubated with the test samples in serial concentration (100 µg/ml to 1000 µg/ml). After 48 h of incubation, cells were washed 2 times with sterile 1XPBS and 100 µl fresh media was added. 10 µl of MTT dye was added to each well and incubated for 4 h (or till colour developed). Live cells change the yellow-coloured MTT dye into the blue to violet colouration. To stop the reaction and dissolution of the coloured product, 100% DMSO was added to each well (Duraipandiyan et al., 2015, Latha et al., 2018).

### 2.8 SEM analysis on *Staphylococcus aureus* in the presence of crude and coumarins-enriched extracts

As the anti-inflammatory property of the enriched extract is targeted in the present study, the inflammatory diseases and the bacteria causing the inflammatory disease should be focussed. *Staphylococcus aureus* is a well-known pathogen which causes severe skin inflammation, showing symptoms like boils, blisters and acne-like rashes. Hence, the effect of the enriched extract was also checked on the bacterium, and SEM analysis was done. *S. aureus* ATCC 6538 culture was revived, and the working culture was prepared by taking one colony from the streaked plate, inoculating in fresh media, and incubating at 37 °C for 24 h. Three sterile test tubes were taken, in which 1 mL bacterial culture (10^8^CFU/mL) was transferred along with 5 mL media; the first one was used as Control, while in the second and third tubes, 0.5 mL of crude and enriched extracts were added at their IC_50_ concentration, as determined by *in vitro* anti-inflammatory activity. All three tubes were sealed and incubated for 48 h at 37 °C. After the incubation completion, the cultures were vortexed and centrifuged at 3000 rpm for 10 min. The supernatant was discarded, and pellets were washed thrice with 1XPBS. Briefly, 200 µl of 2.5% solution of Glutaraldehyde was added to the tubes and incubated at room temperature for 4 h and refrigerated (2–4 °C) for 48 h. After centrifugation, the solution was removed, washed with phosphate buffer thrice, and reconstituted in the same. The vials were sealed and sent to the Sophisticated Analytical Instrumentation Facility, AIIMS, Delhi, for sample viewing (Mishra et al., 2021).

### 2.9 Compound(s) characterization in crude and enriched extract

#### 2.9.1 High-performance thin layer chromatography (HPTLC) analysis

Stock solutions of 0.02 mg/ml for standards viz. marmelosin and marmesin, aegeline, scopoletin, psoralen, and umbelliferone were prepared in methanol. Mobile phase: 12:8 – Hexane: Ethyl acetate; 10 µl of each solution was applied to a 20 cm ×10 cm silica plate with an aluminium coating (Silica gel 60 F_254_) as bands of 10 mm × 2 mm. The mobile phase was allowed to rise 8 cm and examined under ultraviolet light at 254 nm and 366 nm. For quantification, 5 bands of the standard solution prepared were applied serially (1 µl, 2 µl….…0.5 µl) along with one band each of crude extract (5 µl each) and PPI prepared from *Aegle marmelos* fruit powder on a 10 cm × 10 cm plate. The standard curve was plotted, and the standard amount was detected and calculated (Avula et al., 2016).

#### 2.9.2 HPLC analysis

For HPLC analysis, stock solutions of 0.02 mg/ml for standards viz. marmelosin and marmesin, aegeline, scopoletin, psoralen, and umbelliferone and 1 mg/ml solution of crude and enriched extracts were prepared in methanol. 10 µl each of the standard and the test solutions were injected into the HPLC system, Agilent 1260 infinity, connected with a C_18_ column (Orbit) of 150 mm × 4.6 mm and 5 μm pore size. Mobile phase: A. water with 0.1 per cent v/v of glacial acetic acid; B. methanol with 0.1 per cent v/v of glacial acetic acid; column temperature: 35 °C; at 254 nm with gradient program. (Supplementary Table 1).

#### 2.9.3 Direct sample analysis by mass spectrometry (DSA-MS)

The crude and enriched extract of *Aegle marmelos* fruit was subjected to Direct Sample Analyzer MALDI-ToF, Perkin Elmer by putting one drop of sample solution onto the mesh and analyzed in 0–900 *m*/*z* range (Avula et al., 2016).

#### 2.9.4 LC-MS evaluation of crude and enriched extract of *Aegle marmelos* fruit pulp

For LC-MS analysis, the crude and enriched extract (1 mg/ml) was subjected to Agilent LC-MS-ToF; with C_18_ column (Orbit) of 150 mm x 4.6 mm and 5 μm pore size. Mobile phase: A. water with 0.1 per cent v/v of formic acid; B. methanol with 0.1 per cent v/v of formic acid; column temperature: 35 °C; injection volume - 5 µl with the gradient flow described in HPLC method. The obtained peaks were analyzed according to the online MS library on PubChem CID.

#### 2.9.5 ^1^H NMR analysis of crude and enriched extract of *Aegle marmelos* fruit pulp

The crude and enriched extract of *Aegle marmelos* fruit were subjected to ^1^HNMR analysis. 40 mg of the samples were dissolved in 500 μl Deuterated Dimethyl sulfoxide (DMSO) and subjected for ^1^HNMR analysis on Jeol JNM-ECZ 400S, 400 MHz; Pulse program – zg30; Solvent – DMSO-D6; No. of scans – 40; delayed scan – 2; Relaxation time – 4 s; Receiver gain – 101; Acquisition time – 4 s; Pulse width – 16.7 W. Benzoic acid was taken as an internal calibrant. The peaks of the targeted compounds were matched with the individual spectrum of the compounds and integrated accordingly to determine the per cent content of respective compound(s) in the crude and coumarins enriched extracts.

### 2.10 Statistical analysis

All analysis was done in a triplicate (n=3), represented as Mean ± SD. The HPLC software used was EZchrome Elite, WinCats for HPTLC. GraphPad Prism 5 was used for plotting graphs and calculating two-way ANOVA and significance was measured in terms of p-value. For the docking study, Chemdraw, PDB data bank, PubChem, CB-dock, PkCSM, SCF-Bio, Biovia Studio, mgltools and other online support and freeware were used. NMR data was interpreted by using Mestre Nova software.

## 3 Results

### 3.1 Pharmacognostic studies

*Aegle marmelos* fruit pulp powder had 5.78% moisture content, 59.23% water soluble extractive and 35.86% ethanol soluble extractive. It was observed that the fruit contains 2.27% and 0.87% of total ash and acid-insoluble ash, respectively, complying with USP-22. Approximately 22.87% yield of the crude extract while 4.87% yield of coumarins enriched extract was noted. The crude extract showed the presence of alkaloids, phenols, flavonoids, tannins, terpenoids, glycosides, and phytosterols. While only alkaloids, phenols and tannins were detected in enriched extract. (Supplementary Table 2).

### 3.2 *In vitro* anti-hyperglycaemic activity

**Fig. 1:**
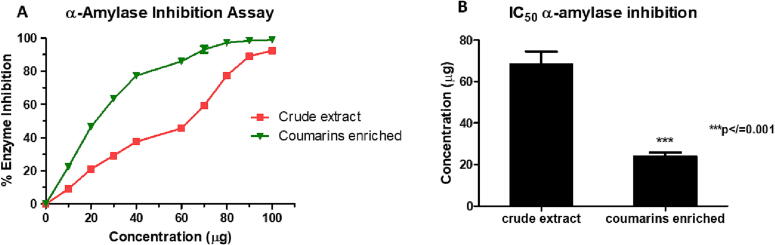
Graphical representation of the α-amylase enzyme inhibition assay shown by crude and enriched *A. marmelos* fruit powder extract. A represents the percentage inhibition curve of both crude and enriched extract. B represents the IC_50_ value of crude and enriched extract of *A. marmelos* fruit. Values expressed as Mean ± SD; n=3; ***p-value ≤ 0.001.

The crude and enriched extract of *Aegle marmelos* fruit showed profound anti-hyperglycaemic activity. The crude extract showed lower activity (IC_50_ 68.35±2.48 µg) compared to the enriched (IC_50_ 23.85±0.78 µg), indicating enrichment of anti-diabetic agents. The enriched extract showed equivalent activity to metformin (IC_50_ value 27.23±0.84 µg) (Supplementary Table 3, Figure 1).

### 3.3 *In vitro* anti-proteinase activity

**Fig. 2:**
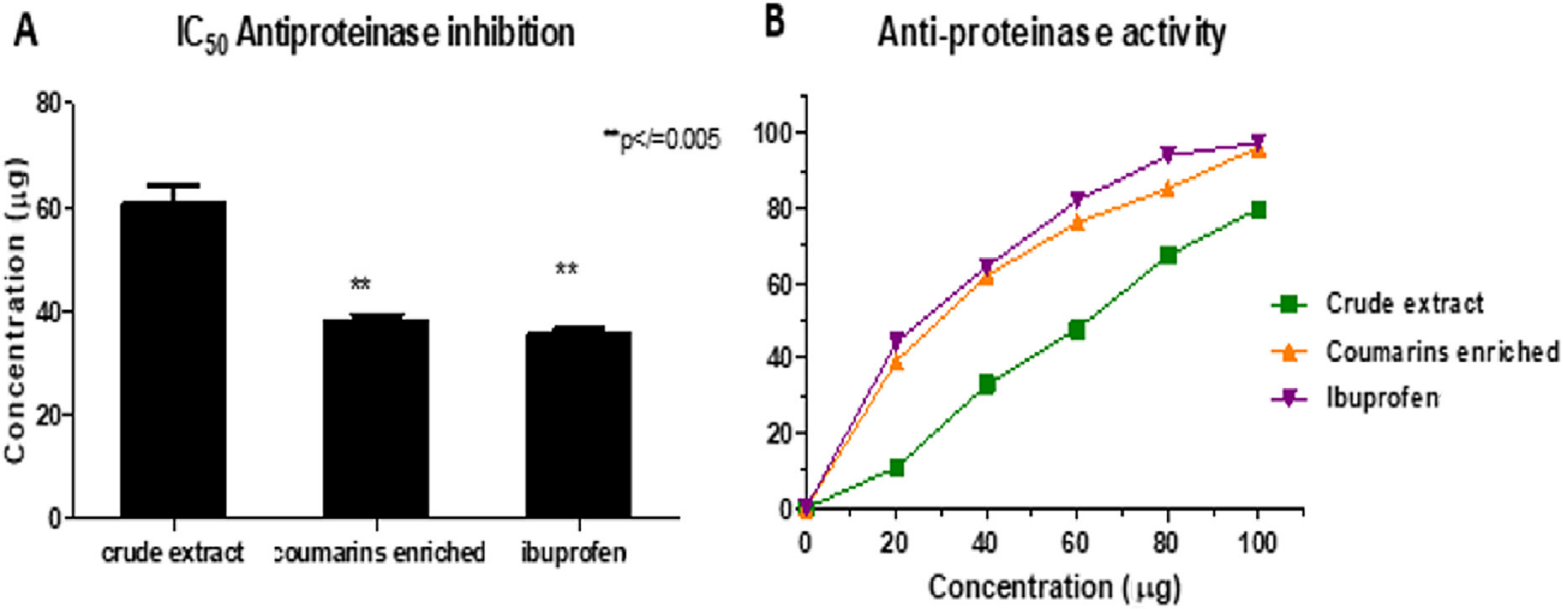
Graphical representation of anti-proteinase assay by showing trypsin enzyme inhibition of crude and enriched extract of *A. marmelos* fruit powder. A represents the IC_50_ value of crude and enriched extract of *A. marmelos* fruit. B represents the percentage inhibition curve of both crude and enriched extract. Values expressed as Mean ± SD; n=3; **p-value ≤ 0.005.

Both crude and enriched extracts exhibited anti-proteinase activity, 79.85% and 96.25% proteinase enzyme inhibition at 100 µg/ml. The anti-proteinase activity comparison was made with ibuprofen, showing 97.45% enzyme inhibition at 100 µg/ml. The IC_50_ value for ibuprofen, the crude and enriched extract was 35.77±0.38 µg, 60.79±1.39 µg and 38.21±0.44 µg, respectively. (Figure 2).

### 3.4 *In silico* studies

#### 3.4.1 Anti-diabetic effect

##### 3.4.1.1 α-amylase inhibitors

**Fig. 3:**
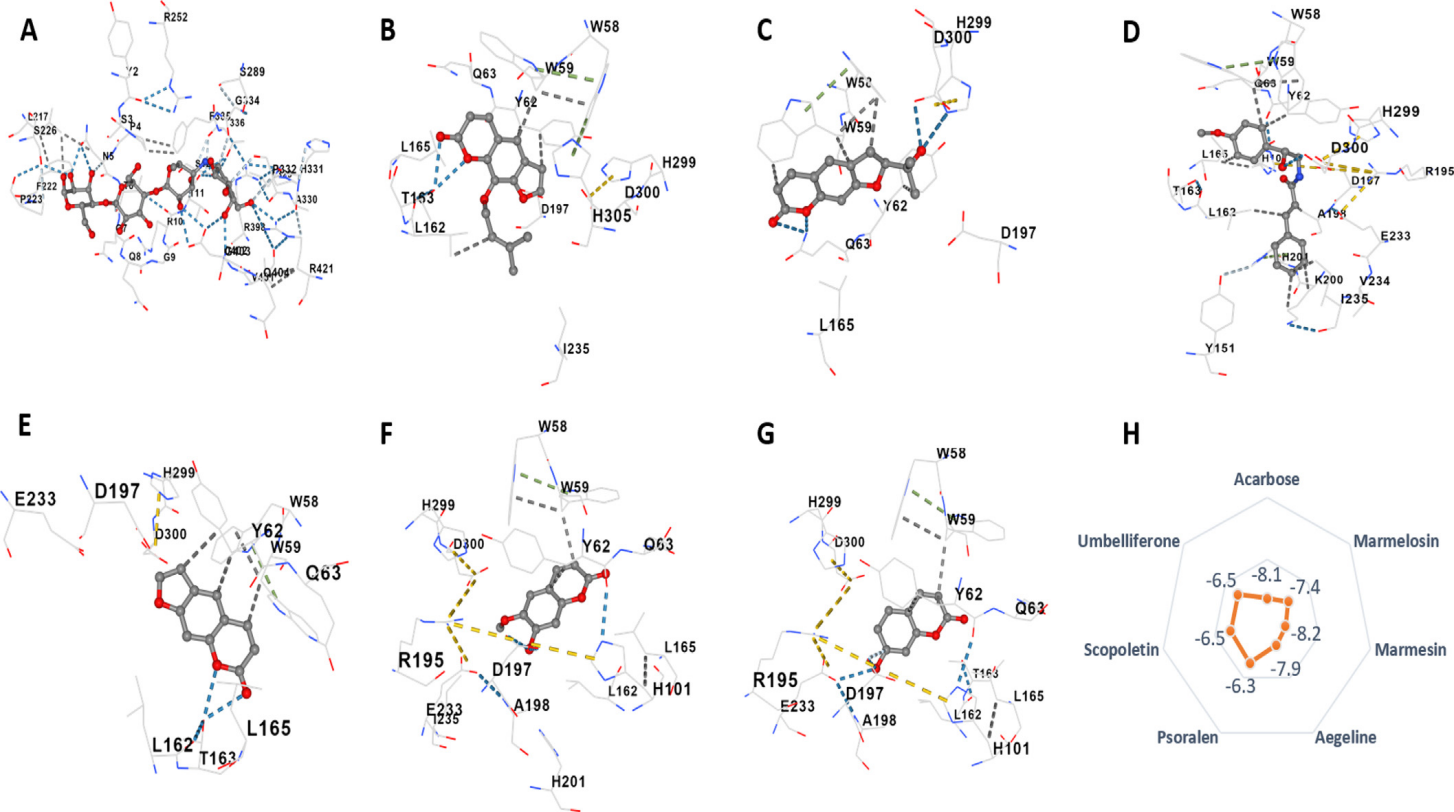
3D molecular interactions with α-amylase enzyme (2QV4) of A) Acarbose, B) Marmelosin, C) Marmesin, D) Aegeline, E) Psoralen, F) Scopoletin, and G) Umbelliferone. H) shows the vina score expressed as kcal/mol of the analytes with the protein binding.

From the evaluation of the molecular docking result on 2QV4 protein, it was found that marmesin showed the highest vina score -8.2 kcal/mol, showed binding on trp58 trp59 tyr62 gln63 leu165 asp197 his299 asp300 residue. Followed by aegeline (-7.9 kcal/mol) and marmelosin (-7.4 kcal/mol). Scopoletin, umbelliferone, and psoralen were found to have the lowest score, -6.5 kcal/mol, -6.5 kcal/mol, and -6.3 kcal/mol, respectively. All compounds bind to non-polar amino acids. Acarbose was found to have a high vina score (-8.1 kcal/mol), comparable to marmesin, aegeline, and marmelosin, binding on non-polar amino acid residues. Binding site of marmesin - trp58 trp59 tyr62 gln63 leu165 asp197 his299 asp300; marmelosin - trp58 trp59 tyr62 gln63 leu162 thr163 leu165 asp197 ile235 his299 asp300 his305; aegeline - trp58 trp59 tyr62 gln63 his101 tyr151 leu162 thr163 leu165 arg195 asp197 ala198 ser199 lys200 his201 glu233 val234 ile235 his299 asp300 his305; psoralen - trp58 trp59 tyr62 gln63 leu162 thr163 leu165 asp197 glu233 his299 asp300; scopoletin - trp58 trp59 tyr62 gln63 his101 leu162 leu165 arg195 asp197 ala198 glu233 ile235 his299 asp300; umbelliferone - trp58 trp59 tyr62 gln63 his101 leu162 thr163 leu165 arg195 asp197 ala198 glu233 his299 asp300; psoralen - trp58 trp59 tyr62 gln63 leu162 thr163 leu165 asp197 glu233 his299 asp300; and acarbose - trp58 trp59 tyr62 gln63 his101 gly104 asn105 ala106 val107 asp147 arg161 leu162 thr163 gly164 leu165 arg195 asp197 ala198 glu233 ile235 asn298 his299 asp300 his305 gly306 (Figure 3; Supplementary Table 4).

##### 3.4.1.2 β-glucosidase

**Fig. 4:**
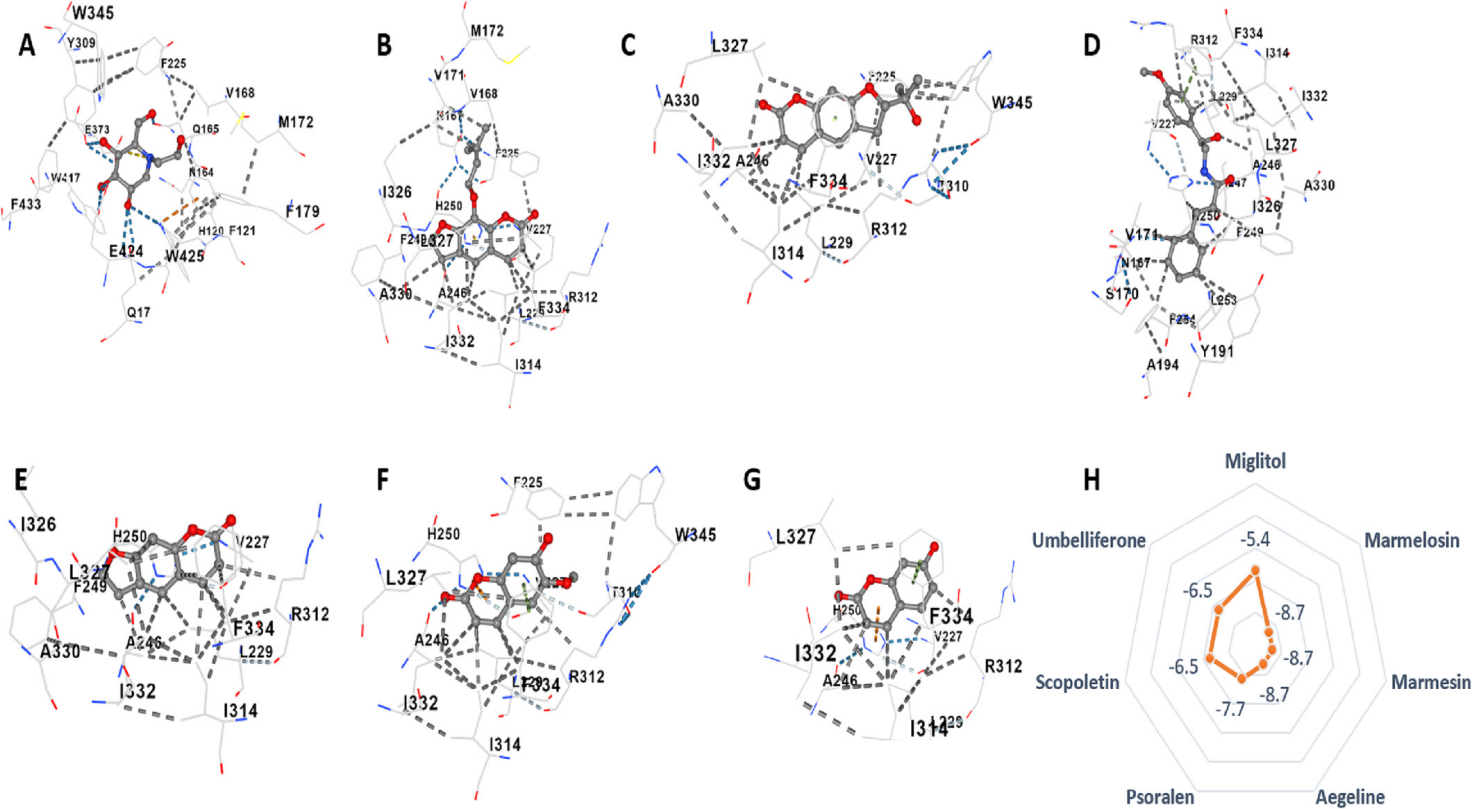
3D molecular interactions with β-glucosidase enzyme (2ZOX) of A) Miglitol, B) Marmelosin, C) Marmesin, D) Aegeline, E) Psoralen, F) Scopoletin, and G) Umbelliferone. H) shows the vina score expressed as kcal/mol of the analytes with the protein binding.

All the target analytes and the standard drug miglitol were targeted on the β-glucosidase enzyme 2ZOX protein. It was noted that marmelosin, marmesin, and aegeline showed the highest binding score, i.e., -8.7 kcal/mol. While the standard drug miglitol, which is very commonly used for type 2 diabetes treatment, showed a -5.4 kcal/mol binding score on the enzyme, the lowest among all analytes. Besides this, scopoletin, umbelliferon, and psoralen had binding scores of -6.5 kcal/mol, -6.5 kcal/mol, and -7.7 kcal/mol, respectively. This shows the great potential of coumarins as an anti-diabetic agent. Binding site of marmelosin was found to be asn167 val168 val171 met172 val227 leu229 ala246 phe249 his250 arg312 ile314 ile326 leu327 ala330 ile332 phe334; binding site of marmesin was found to be phe225 val227 leu229 ala246 thr310 arg312 ile314 leu327 ala330 ile332 phe334 trp345; aegeline - asn167 ser170 val171 tyr191 ala194 val227 leu229 ala246 phe249 his250 leu253 phe254 thr310 arg312 ile314 ile326 leu327 ala330 ile332 phe334 trp345; scopoletin - val227 leu229 ala246 his250 thr310 arg312 ile314 leu327 ile332 phe334 trp345; psoralen - val227 leu229 ala246 phe249 his250 arg312 ile314 ile326 leu327 ala330 ile332 phe334; umbelliferone - val227 leu229 ala246 his250 arg312 ile314 leu327 ile332 phe334; and miglitol - gln17 his120 phe121 asn164 gln165 val168 met172 phe179 phe225 tyr309 trp345 glu373 trp417 glu424 trp425 phe433. (Figure 4; Supplementary Table 4).

##### 3.4.1.3 Pancreatic lipase inhibitors

**Fig. 5:**
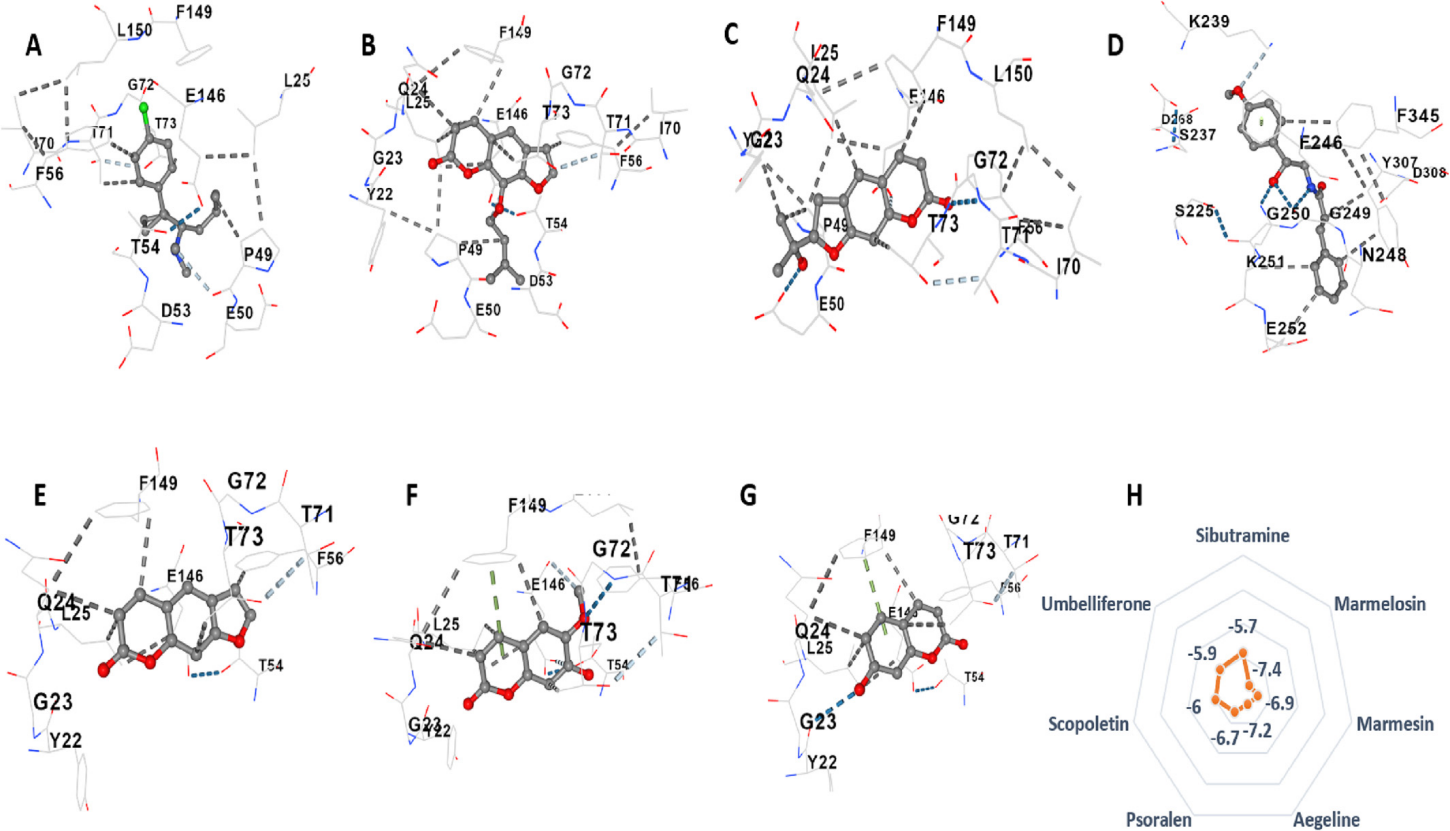
3D molecular interactions with human pancreatic lipase (2OXE) of A) Sibutramine, B) Marmelosin, C) Marmesin, D) Aegeline, E) Psoralen, F) Scopoletin, and G) Umbelliferone. H) shows the vina score expressed as kcal/mol of the analytes with the protein binding.

Molecules were also targeted to the 2OXE enzyme (human pancreatic lipase), where sibutramine was used as the standard drug. It was observed that marmelosin showed the best binding score (-7.4 kcal/mol), followed by aegeline (-7.2 kcal/mol), marmesin (-6.9 kcal/mol), and psoralen (-6.7 kcal/mol). Scopoletin and umbelliferone showed lower scores -6.0 kcal/mol and -5.9 kcal/mol, respectively; however, the standard drug sibutramine showed the lowest vina score of -5.7 kcal/mol, which again proves the coumarins are a better substitute to the existing lipase inhibitors indicating anti-diabetic potential. The binding amino acid residue of scopoletin was found to be phe91 gln92 his94 his96 glu106 his119 ala121 leu131 ala135 leu141 val143 leu198 thr199 his200 pro201 pro202 trp209, and for umbelliferone, it was phe91 his94 his96 glu106 his119 ala121 leu141 val143 leu198 thr199 his200 pro202 trp209. Marmelosin and triamterene had the same binding site and size coordinates (48,21,–23) and (20,20,20), respectively. Both molecules were found to interact with non-polar residue viz.; pro3 trp5 gly6 tyr7 asp8 asn11 val62 gly63 his64 ser65 lys170 ser231 val239 pro240 met241 gln242 his243 asn244; and trp5 gly6 tyr7 asp8 asn11 val62 gly63 his64 ser65 lys170 ser231 val239 pro240 met241 gln242 his243 asn244 respectively (Figure 5; Supplementary Table 4).

#### 3.4.2 Anti-inflammatory effect

**Fig. 6:**
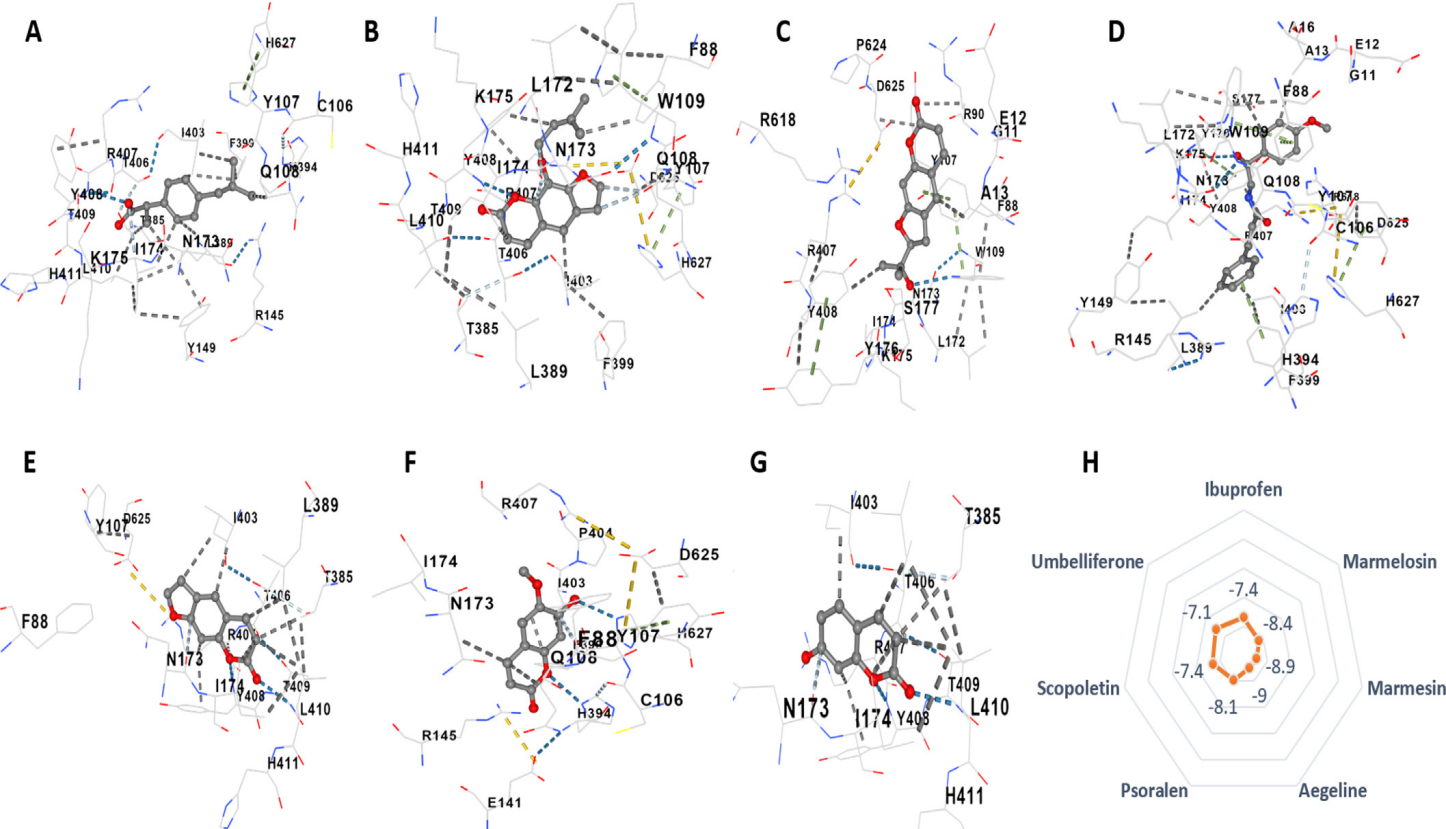
3D molecular interactions with LOX2 of A) Ibuprofen, B) Marmelosin, C) Marmesin, D) Aegeline, E) Psoralen, F) Scopoletin, and G) Umbelliferone. H) shows the vina score expressed as kcal/mol of the analytes with the protein binding.

LOX-2 protein was studied to determine the anti-inflammatory effect of the active molecules of *Aegle marmelos*. It was found that aegeline, marmelosin and marmesin showed the highest docking score (-9.0 kcal/mol, -8.4 kcal/mol and -8.9 kcal/mol, respectively), followed by psoralen (-8.1 kcal/mol), scopoletin (-7.4 kcal/mol) and umbelliferone (-7.1 kcal/mol). Aegeline, marmelosin, and marmesin have outperformed the standard anti-inflammatory drug ibuprofen, which had a docking score of -7.4 kcal/mol. Marmelosin was found to bind cys106 tyr107 gln108 arg145 tyr149 asn173 ile174 thr385 leu389 his394 phe399 ile403 thr406 arg407 tyr408 thr409 leu410 asp625 his627 amino acid residue; aegeline binds on phe184 phe365 glu369 his373 leu374 his378 ile412 asn413 leu415 ala416 leu419 leu420 val427 phe438 gln560 leu607 leu610 ile676 residue; and marmesin binds on gly11 glu12 ala13 phe88 arg90 trp109 leu172 asn173 ile174 lys175 tyr176 ser177 arg407 tyr408 arg618 pro624 asp625. Psoralen was found to interact with tyr107 asn173 ile174 thr385 leu389 ile403 thr406 arg407 tyr408 thr409 leu410 his411 asp625, while scopoletin was shown to interact with tyr107 asn173 ile174 lys175 thr385 leu389 ile403 thr406 arg407 tyr408 thr409 leu410 his411. Umbelliferone and ibuprofen were binding with asn173 ile174 lys175 thr385 leu389 ile403 thr406 arg407 tyr408 thr409 leu410 his411 asp625 and cys106 tyr107 gln108 asn173 ile174 lys175 thr385 leu389 his394 phe399 ile403 thr406 arg407 tyr408 thr409 leu410 his411 his627 chains respectively. The above result shows that the targeted coumarins have anti-inflammatory potential, with marmelosin, marmesin, and aegeline being the best ones (Figure 6; Supplementary Table 4).

#### 3.4.3 ADMET study

**Fig. 7:**
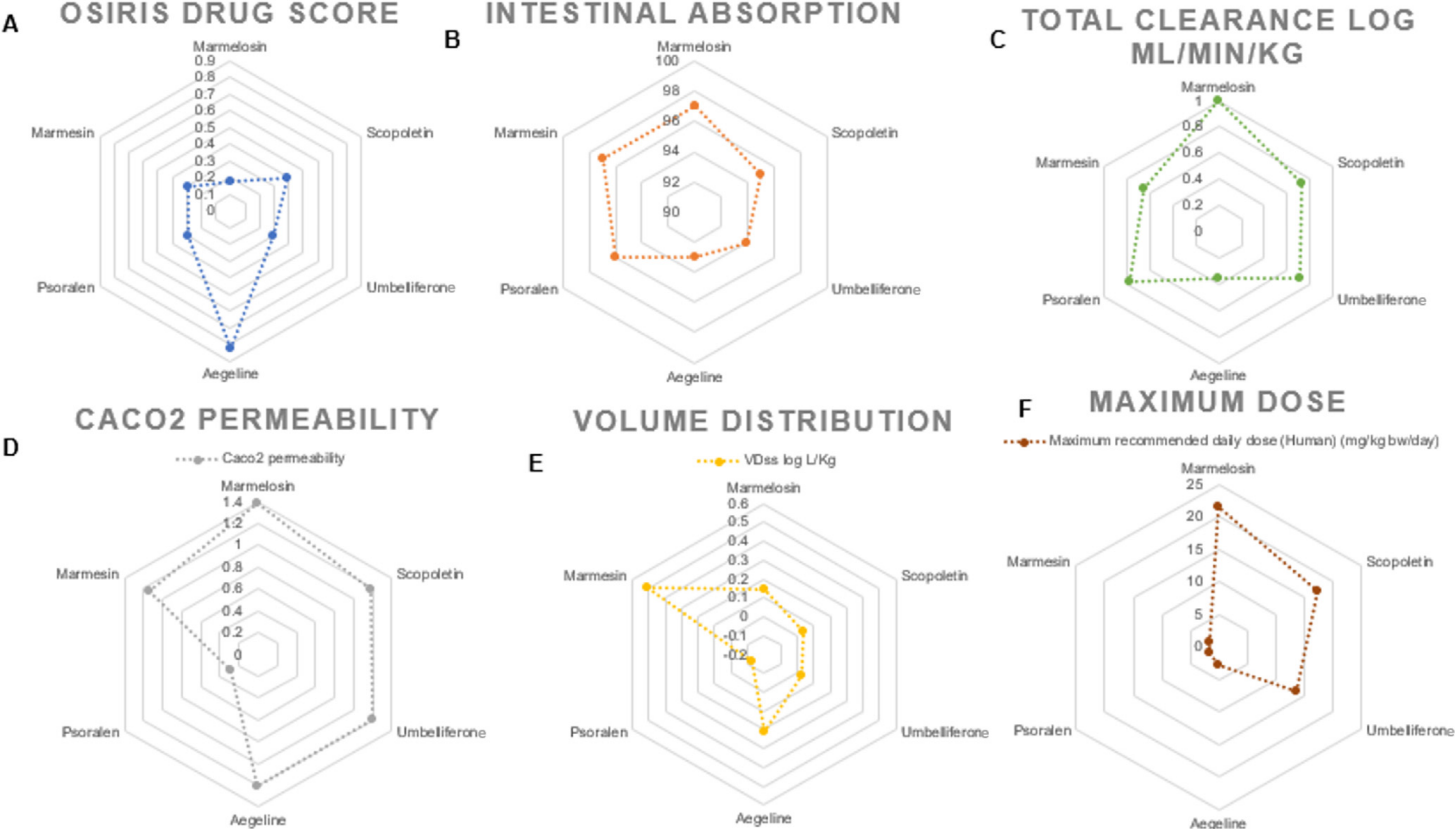
ADMET study of all coumarins targeted for enrichment in *A. marmelos* fruit extract. A) represents drug-likeness score, B) represents intestinal absorption of molecules, C) represents total renal clearance, D) represents molecules absorption in colon, E) represents volume distribution of the target analyte, and F) represents maximum daily dose recommendation for human beings per kg body weight.

All targeted molecules followed Lipinski's rule of 5, making them suitable drug candidates. Marmelosin showed the highest intestinal absorption with no hepatotoxicity and a high clearance rate. However, the compound was observed to have low OSIRIS drug-likeness and VDss scores, indicating a lower drug volume distribution. Although it was found to be the substrate of CYP3a4, it is non-inhibitory, showing its degradation by mitochondrial enzymes. Similarly, the molecules were found to be easily permeable to the skin and also through colon cells. All molecules except aegeline were found to be non-mutagenic, whereas umbelliferone was found to be hepatotoxic. The maximum recommended daily dose of marmelosin was found to be 21.5 mg/kg bw/day. While scopoletin and umbelliferone were predicted to be 17.4 mg/kg bw/day and 13.6 mg/kg bw/day, respectively. In the case of aegeline, psoralen, and marmesin, the dosage was found to be 2.68 mg/kg bw/day, 1.6 mg/kg bw/day, and 1.61 mg/kg bw/day, respectively (Figure 7; Supplementary Table 5).

### 3.5 *In vitro* cell cytotoxicity assay

**Fig. 8:**
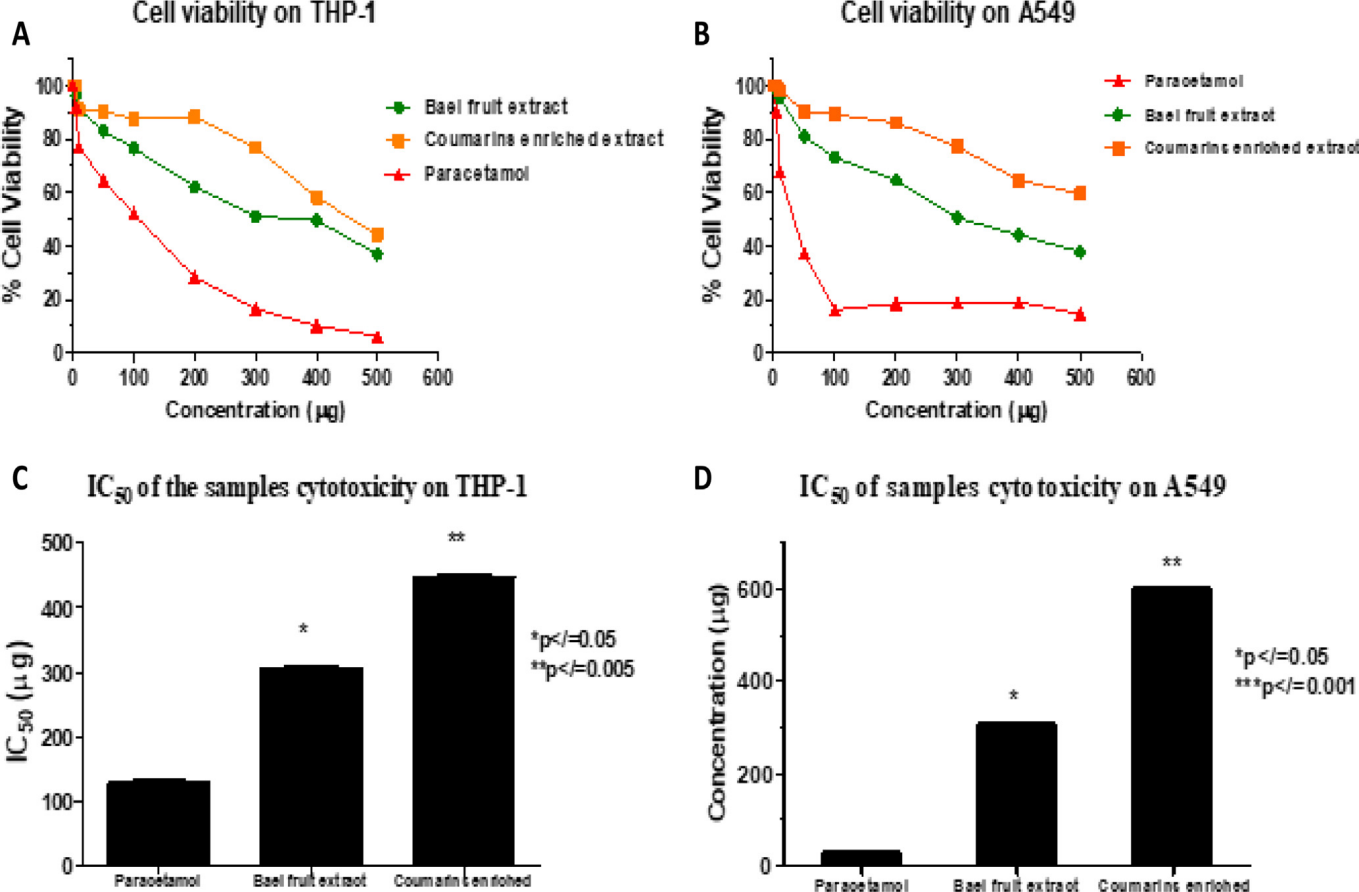
Graphical representation of cell cytotoxicity assay of *A. marmelos* fruit crude and enriched extracts in terms of % cell viability on (A) THP-1 and (B) A549 cell lines. C and D represent the bar graph representation of the IC_50_ value of *A. marmelos* fruit crude and enriched extracts on (C) THP-1 and (D) A549 cell lines. Samples were compared with the standard anti-inflammatory drug – Paracetamol. Values expressed as Mean ± SD; n=3; *p-value≤0.05; **p-value≤0.005; ***p-value≤0.001.

From the MTT assay conducted on the two cell lines viz., THP-1 and A549, cytotoxicity of the crude and enriched extracts was significantly lower than the standard NSAIDs drug – Paracetamol. The crude extract was more cytotoxic than the coumarins-enriched extract, confirming the ADMET predictions. It was found that the IC_50_ of paracetamol on both cell lines were the lowest (129.50 ± 0.97 µg and 28.50 ± 2.23 µg for THP-1 and A549 cell lines, respectively), which makes it more cytotoxic than the crude (306.18 ± 2.83 µg and 448.39 ± 1.76 µg for THP-1 and A549 cell lines respectively) and enriched extracts (336.18 ± 1.38 µg and 618.26 ± 0.79 µg for THP-1 and A549 cell lines respectively). The difference between the IC_50_ value of the standard drug and the extracts is very significant according to the two-way ANOVA test conducted between the samples and the standard. Moreover, paracetamol was more toxic for A549 cell lines with IC_50_ concentration lower than on THP-1 cell lines. Coumarins enriched extract exhibited lower cytotoxic on A549 cell lines as its IC50 value is higher on A549 than THP-1. The crude extract of *Aegle marmelos* fruit was found to have an almost equivalent effect on both cell lines, as observed from its IC_50_ value. (Figure 8).

### 3.6 SEM analysis on *S. aureus* in the presence of crude and enriched extracts

**Fig. 9:**
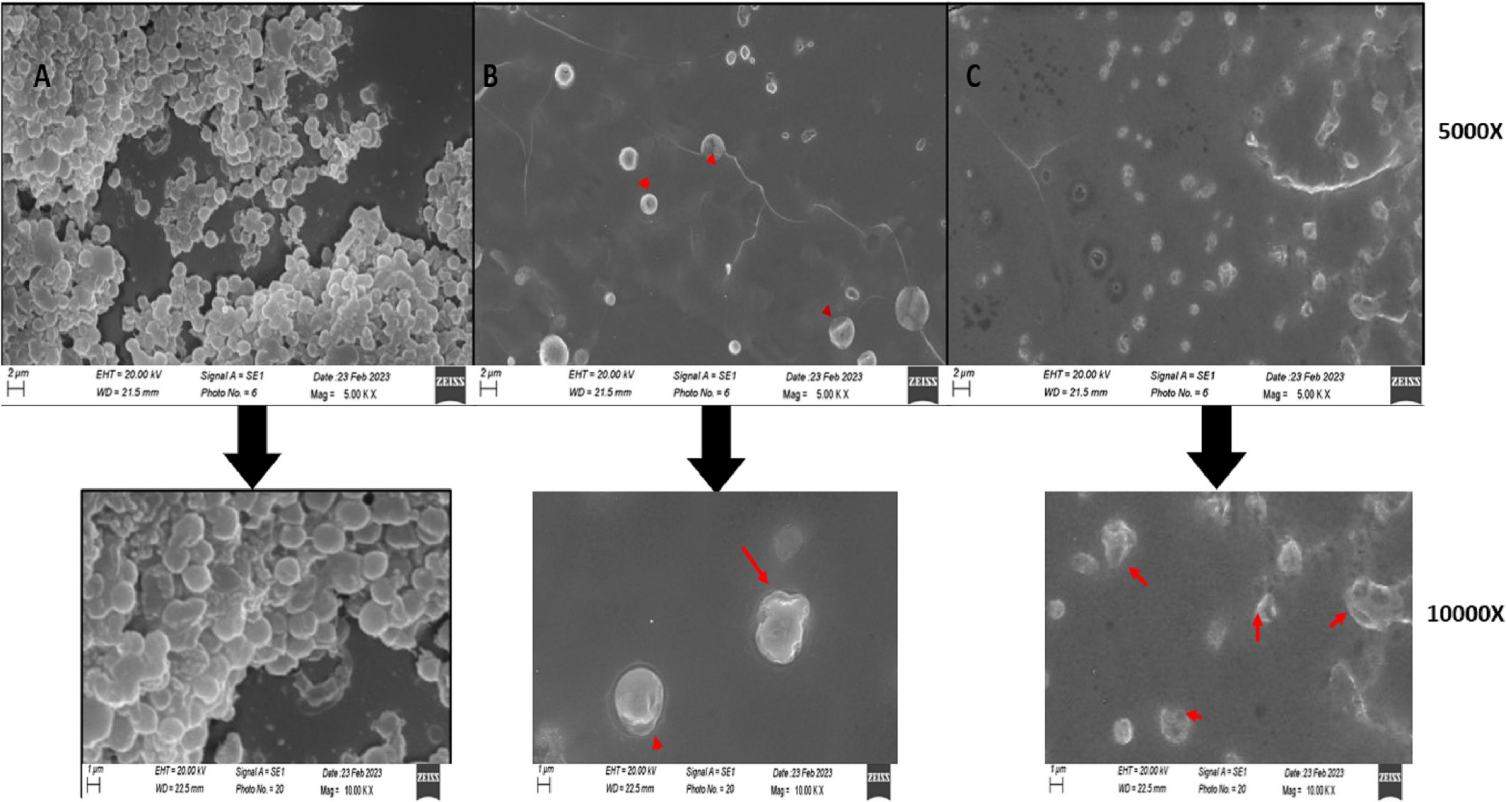
Pictorial representation of SEM analysis on *S. aureus* in the presence of crude extract and coumarins-enriched extracts of *A. marmelos* fruit at 5000X and 10000X magnification. (A) represents healthy cells growing in the absence of any inhibitory agent. (B) represents bacterial cells growing in the presence of the crude extract of *A. marmelos* fruit. (C) represents bacterial cells growing in the presence of the coumarins-enriched extract of *A. marmelos* fruit. Red arrows show the bacterial cells with visible cell wall damage.

From the SEM analysis, crude and coumarins-enriched extracts of *Aegle marmelos* fruit showed an anti-bacterial effect on *S. aureus*. In the control sample, the bacteria were growing in clusters; in the presence of test samples, growth was inhibited and isolated. In the presence of crude extracts, some cells were intact, but most had disfigured cell walls, and holes with cytoplasmic leakage, confirming the cell death. In the presence of enriched extract, all cells were found dead, necrotic, with heavy cell wall damage, as observed at 10,000 magnifications, confirming the antibiotic nature of the enriched extract. This also shows that the enriched extract may have a bactericidal effect as it is causing permanent damage to the cells, hence having a high probability of being used as prescription medicine in upcoming years (Figure 9).

### 3.7 Compound(s) characterization and quantification in crude and enriched extract

#### 3.7.1 HPTLC analysis

From the HPTLC analysis of the crude and enriched extract of *Aegle marmelos* fruit powder, all six bioactive compounds viz.; marmelosin, marmesin, aegelin, psoralen, umbelliferone, and scopoletin were detected. Marmelosin was detected at Rf 0.65, followed by psoralen (Rf 0.62), umbelliferone (Rf 0.58), scopoletin (Rf 0.36), aegeline (Rf 0.34), and marmesin (Rf 0.30). It is to be noted that marmelosin, marmesin, aegeline, and scopoletin are visible as dark spots at 254 nm. Whereas umbelliferone is visible only at 366 nm as it fluoresces at that wavelength. All the standards, except aegeline, are visible at 366 nm. The crude and enriched extracts have marmelosin in the highest content at 2.93% and 27.65%, respectively. Apart from this, 2.37% marmesin, 0.24% aegeline, 2.06% psoralen,1.18% scopoletin, and 1.28% umbelliferone were found in crude extract, while enriched extract was found to contain 5.12% marmesin, 0.67% aegeline, 3.83% psoralen, 3.13% scopoletin, and 1.92% umbelliferone (Supplementary Table 6; Supplementary Figure 3).

#### 3.7.2 HPLC analysis

The HPLC analysis has confirmed the findings of the HPTLC result. The standards viz.; marmelosin, marmesin, aegeline, psoralen, scopoletin, and umbelliferone in the crude and enriched extracts were detected at 10.4, 5.4, 7.2, 5.9, 4.2, and 3.4 min, respectively. The relative retention time of all standards was found to be not more than 0.5 min. The crude extract was found to contain 2.84% marmelosin, 3.95% marmesin, 0.13% aegeline, 2.35% psoralen, 1.52% scopoletin, and 1.44% umbelliferone. The coumarins-enriched extract was found to have 31.20% marmelosin, 8.89% marmesin, 0.72% aegeline, 4.06% psoralen, 2.03% scopoletin, and 1.71% umbelliferone (Supplementary Table 7; Supplementary Figure 4).

#### 3.7.3 DSA-MS

The abundance of molecular peak ion intensity can be seen raised in the *Aegle marmelos* coumarins enriched extract compared to the crude extract. Marmelosin was characterized at 280.2358 (M+10H^+^), 334.3005 (2 M+Na+ACN), and 335.3079 (2 M+Na+ACN+H^+^), along with marmesin at 180.1543 (M/2+Na^+^), 181.1509 (M/2+Na^+^+H^+^), and 182.1722 (M/2+Na^+^+2H^+^). Minute peaks of aegeline were also detected at 306.2331 (M+Na^+^), 307.2489 (M+Na^+^+H^+^), and 308.2582 (M+Na^+^+2H^+^). Peaks corresponding to scopoletin, psoralen, and umbelliferone were 198.1689 (M+6H^+^) and 200.1849 (M+8H^+^); 335.295 (2 M+Na^+^+2H^+^); and 855.98785 (5 M+2Na^+^), 872.97083 (5 M+K^+^+Na^+^), and 873.05446 (5 M+K^+^+Na^+^) respectively (Supplementary Table 8).

#### 3.7.4 LC-MS evaluation

The LC-MS results have reconfirmed the DSA-MS results as both samples showed one major peak around 2–2.5 min (2.247 min in crude extract and 2.306 min in enriched extract), which was analyzed and found to contain marmelosin as the principal molecule, giving molecular peak at 271.1066 (M+H^+^), 272.10196 (M+2H^+^), and 102.12638 (M/3+H^+^). Marmesin was detected at 288.13124 (M+ACN) and 289.12551 (M+ACN+H^+^); psoralen at 715.30576 (3 M+2ACN+Na^+^+K^+^); scopoletin at 383.18844 (2 M^+^) and 388.40061 (2 M+4H^+^); and umbelliferone at 558.2228 (3 M+ACN+Na^+^+7H^+^), and 559.22143 (3 M+ACN+Na^+^+8H^+^). Only crude extract showed aegeline presence at 350.16733 (M+2Na^+^+7H^+^) (Supplementary Table 9; Supplementary Figure 5).

#### 3.7.5 NMR analysis and quantification of crude and enriched extract

^1^H NMR spectrum of crude and the enriched extract was found to have signature peaks of all six bioactive compounds. The ^1^H NMR peak lists of crude extract, PPI, and standards are: - Marmelosin: *-*mol. weight – 270.28; ^1^HNMR (400 MHz, DMSO-D_6_, 2.47) *δ* 8.11 (s), 8.09 – 8.07 (m), 7.64 (s), 7.05 (d, *J* = 2.2 Hz), 6.39 (d, *J* = 9.6 Hz), 5.50 – 5.43 (m), 4.87 (d, *J* = 7.2 Hz), 1.62 (d, *J* = 20.8 Hz), 1.18 (s). Marmesin: *-* mol. weight – 246.26; ^1^H NMR (500 MHz, DMSO-D_6_, 2.47) *δ* 7.70 (d, *J* = 10.8 Hz, 1H), 7.32 (s, 1H), 6.36 (d, *J* = 11.0 Hz, 1H), 5.05 (s, 1H), 4.78 (s, 1H), 3.99 (t, *J* = 8.7 Hz, 1H), 3.49 (s, 1H), 3.41 (dd, *J* = 12.5, 8.6 Hz, 1H), 1.88 (dd, *J* = 12.4, 8.7 Hz, 1H), 1.34 (s, 5H). Aegeline: - mol. weight –297.3; ^1^H NMR (500 MHz, DMSO-D_6_, 2.47) *δ* 7.59 – 7.47 (m, 3H), 7.45 – 7.32 (m, 3H), 7.26 (dd, *J* = 7.5, 1.4 Hz, 1H), 6.98 (dd, *J* = 7.5, 1.4 Hz, 1H), 6.61 – 6.48 (m, 2H), 5.77 (dd, *J* = 7.5, 1.4 Hz, 1H), 4.85 (s, 1H), 4.49 (t, *J* = 2.2 Hz, 1H), 3.80 (s, 3H), 3.62 (dd, *J* = 12.5, 2.2 Hz, 1H), 3.41 – 3.33 (m, 2H). Psoralen: - mol. weight – 186.16; ^1^HNMR (400 MHz, DMSO-D_6_, 2.47) *δ* 8.14 (d, *J* = 9.6 Hz), 8.08 (d, *J* = 2.3 Hz), 7.98 (s), 7.70 (s), 7.07 (dd, *J* = 2.3, 1.0 Hz), 6.40 (d, *J* = 9.6 Hz), 2.04 (s). Scopoletin: - mol. weight – 192.17; ^1^HNMR (400 MHz, DMSO-D_6_, 2.47) *δ* 7.87 (d, *J* = 9.5 Hz), 7.18 (s), 6.74 (s), 6.18 (d, *J* = 9.5 Hz), 3.77 (s). Umbelliferone: - mol. weight – 162.14; ^1^HNMR (400 MHz, DMSO-D_6_, 2.47) *δ* 10.59 (s), 7.89 (d, *J* = 9.4 Hz), 7.49 (d, *J* = 8.5 Hz), 6.86 – 6.57 (m), 6.16 (d, *J* = 9.5 Hz), 2.04 (s). Multiplets found in a crude extract of *Aegle marmelos* fruit pulp: *-* ^1^HNMR (400 MHz, DMSO-D_6_, 2.47) *δ* 8.03 (dd, *J* = 14.1, 9.8 Hz), 7.93 (s), 7.90 (s), 7.88 – 7.83 (m), 7.68 (d, *J* = 8.3 Hz), 7.57 (s), 7.56 – 7.50 (m), 7.41 (dd, *J* = 11.3, 4.1 Hz), 7.31 (s), 6.96 (d, *J* = 2.2 Hz), 6.54 (s), 6.32 (d, *J* = 9.6 Hz), 6.18 (d, *J* = 9.6 Hz), 5.41 – 5.35 (m), 5.25 – 5.21 (m), 5.13 (d, *J* = 3.6 Hz), 4.89 (d, *J* = 3.5 Hz), 4.81 (d, *J* = 7.2 Hz), 4.26 (d, *J* = 7.7 Hz), 4.08 (s), 3.39 (qd, *J* = 7.1, 0.9 Hz), 2.47 (s), 1.54 (d, *J* = 24.2 Hz), 0.99 (td, *J* = 7.0, 0.9 Hz). Multiplets found in the enriched extract of *Aegle marmelos* fruit pulp: - ^1^HNMR (400 MHz, DMSO-D_6_, 2.47) *δ* 8.13 (d, *J* = 5.7 Hz), 8.07 (dd, *J* = 11.2, 5.9 Hz), 7.96 (s), 7.90 (dd, *J* = 8.2, 1.0 Hz), 7.75 (d, *J* = 8.6 Hz), 7.69 (s), 7.63 (s), 7.60 – 7.54 (m), 7.45 (t, *J* = 7.6 Hz), 7.34 (s), 7.04 (d, *J* = 2.2 Hz), 6.38 (d, *J* = 9.6 Hz), 6.23 (d, *J* = 9.5 Hz), 5.49 – 5.43 (m), 5.15 (d, *J* = 3.7 Hz), 4.89 – 4.85 (m), 1.62 (d, *J* = 20.3 Hz). The per cent content of marmelosin calculated by NMR was 2.12% and 29.25% in crude and enriched extract, respectively. In the crude extract of *Aegle marmelos*, the content of marmesin, aegeline, psoralen, scopoletin, and umbelliferone was detected at 2.34%, 0.07%, 1.21%, 0.85% and 0.89% respectively. While in the enriched extract, the content of marmesin, aegeline, psoralen, scopoletin, and umbelliferone was detected to be 6.01%, 0.3%, 4.44%, 2.61% and 1.13%, respectively (Supplementary Table 10; Supplementary Figure 6a&b).

## 4 Discussion

Since prehistoric times, humans have been utilizing and repurposing medicinal plants for disease treatment (Wachtel-Galor and Benzie, 2011). With time and the uprisal of modern medicines armed with science and technology, several synthetic drugs have overshadowed traditional knowledge (Singh, 2010). Although causing specific pharmacological effects, synthetic drugs also result in some unavoidable adverse effects. Hence, researchers are moving towards herbal medicines and formulations in the current scenario (Joshi et al., 2011, Sen and Chakraborty, 2017). The sudden increase in the demand for herbal medicines raises issues regarding their safety and efficacy, impeding their acceptability by Indian Medical Association (IMA) and at the global level as mainstream medicines (Ekor, 2013, Katiyar, 2019). The global acceptability for herbal medicines can only be reached if these are developed scientifically. To fulfil this purpose, the Government of India has designed a concept called “Phytopharmaceutical drugs.” Phytopharmaceutical drugs include the purified fraction(s) of the medicinal plant or its part with scientifically proven bioactivity (Bhatt, 2016, Katiyar, 2019). The purified fraction of the plant or its part is considered as a phytopharmaceutical drug if quantification and characterization of a minimum of four marker compounds are done. The chosen marker compounds should contain at least one bioactive marker with defined pharmacological activity (Katiyar, 2019). Our research study is based on the above-mentioned concept, providing a scaffold for phytopharmaceutical drug development.

The present study has developed the coumarins enrichment process in *Aegle marmelos*, which has successfully enriched bioactive compounds such as marmelosin, marmesin, aegeline, psoralen, scopoletin, and umbelliferone. The method developed is simple, sophisticated and economical, which can easily be implemented at the industrial level. Several research studies have been carried out regarding the isolation of coumarins, which are known bioactive compounds in *Aegle marmelos* (Chakthong et al., 2012, Manandhar et al., 2018, Shinde and Laddha, 2012, Tiwari et al., 2020). However, the methodologies described earlier for the coumarins compound(s) isolation are complicated and cannot be applied at a large scale (Chakthong et al., 2012). We have depicted and developed a simple liquid–liquid partition technique combined with VLC, easing the separation process with a good percentage yield. The total coumarins were isolated in three easy steps. First, crude extract preparation from *Aegle marmelos* fruit pulp; second, liquid–liquid partitioning of the aqueous solution of the concentrated methanolic extract with ethyl acetate; and third, separation of the coumarins-enriched extract by VLC.

Previously, several *in vitro* and *in vivo* studies have been conducted recurringly on different *Aegle marmelos* fruit extracts. Out of these, many emphasize the anti-diabetic and anti-inflammatory activities of the plant fruit (Arul et al., 2005a, Arul et al., 2005b, Kesari et al., 2006, Rajeshkannan et al., 2014, Sabu and Kuttan, 2004, Sudharameshwari and Radhika, 2007, Vyas et al., 2011). The present study has demonstrated the anti-diabetic and anti-inflammatory activities of the coumarins-enriched extract isolated from the *Aegle marmelos* fruit, accentuating their potential use. It was found that the coumarins-enriched extract isolated from the *Aegle marmelos* fruit greatly inhibits α-amylase and proteinase enzymes, indicating a potent anti-diabetic and anti-inflammatory agent. The bioactivity of the enriched extract was greatly enhanced when compared with the crude extract emphasizing the isolation of potent bioactive components. The study also proves its activity is significantly better than the renowned anti-inflammatory and hypoglycemic drugs – ibuprofen and metformin. The difference in the IC_50_ value of the enriched extract and the standard(s) was found to be significant (p-value ≤ 0.01).

Our research further elaborates *in silico* molecular docking of three main proteins: - α-amylase, β-glucosidase, and pancreatic lipase, which plays a key role in upregulating diabetic symptoms. A total of six coumarins, along with the standard drugs specific for the inhibition of the enzymes, were docked. The results showed that the coumarins of *Aegle marmelos* have a better affinity with the enzymes than that of the standard anti-diabetic drug. It was observed that the pocket volume in the case of α-amylase enzyme, for the coumarins were larger compared to acarbose indicating a lock and key type of interaction. While for β-glucosidase and pancreatic lipase enzymes, the pocket size is smaller than miglitol and sibutramine respectively, from which we can surmise that they may have better affinity and the interaction might be induced-fit type (Chen et al., 2014, Gao and Skolnick, 2012, Meng et al., 2011).

The NSAIDs used for treating inflammation are Cyclooxygenase (COX) inhibitors (Lehmann and Beglinger, 2005, Zarghi and Arfaei, 2011). Some of these specifically inhibit the COX-2 enzyme. COX enzymes are known to produce proinflammatory products, which leads to inflammation. Although the enzyme is known to act upon the chronic inflammation caused due to the diseases, it is also known to cause heart stroke by creating prostanoids imbalance in the renal blood (Sharma and Jawad, 2005, Wright, 2002). Therefore, we chose a new target proinflammatory enzyme – LOX-2, which limits the metabolism of arachidonic acid into leukotriene, mediating inflammation. The enzyme is also known to be actively involved in chronic respiratory disease, atherosclerosis and cancer (Dobrian et al., 2011, Fiorucci et al., 2001, Hu and Ma, 2018, Natarajan and Nadler, 2004). Hence, the coumarins of *Aegle marmelos* were docked along with the standard NSAID ibuprofen on LOX-2 enzyme. Our *in silico* docking results showed a very high affinity for the enzyme, with active binding size, indicating an induced-fit interaction. The binding energy of standard NSAID was found higher binding energy, showing lower affinity, hence poor inhibition. Overall, all the coumarins have outperformed the standard anti-diabetic and anti-inflammatory drugs – indicating themselves as promising lead compounds. Their cumulative effect will be synergistic and therefore are precursors for phytopharmaceutical ingredients development.

Further, we have analyzed the *in silico* ADMET effect as well as the *in vitro* cytotoxicity evaluation to elucidate adverse reactions (if any) caused by the compounds targeted for enrichment from *Aegle marmelos* fruit. It was observed that the six coumarins present in the enriched extract, showed overall good absorption, but low volume distribution. The molecules have no cytotoxic effects with excellent skin permeability, which indicates that the gel formulation containing the compounds should work effectively. A slight precaution is needed concerning the content of aegeline and umbelliferone, which showed mutagenic and hepatotoxic effects at higher dosage. This is to be noted that in the enrichment process developed in our study, the content of aegeline and umbelliferone was detected to be 0.72 % w/w and 1.71 % w/w only. Hence, the method developed for coumarins enrichment is significant for industrial purposes. The *in silico* ADMET results predictions have been confirmed by *in vitro* cytotoxic assay carried out on the THP-1 and A549 cell lines. The enriched extract of *Aegle marmelos* was found non-toxic as the IC_50_ on these cell lines was found to be more than 500 µg, while for the NSAIDs, it was less than 100 µg, which is significantly higher. This indicates the non-toxic nature of the coumarins-enriched extract.

Diabetes is known to cause chronic inflammation as well as weaken the immune system of the patient, facilitating several health complications (Guthrie and Guthrie, 2004, Kaveeshwar and Cornwall, 2014, Olokoba et al., 2012, Varsha et al., 2018, Wilson, 2007). The increased risk in infection during diabetes is commonly observed in the individual as skin and soft tissue infection (SSTI) (Suaya et al., 2013, Zhou and Lansang, 2021). The causative agent for the infection is *Staphylococcus aureus* (Thurlow et al., 2020). Therefore, the enriched extract was studied with *S. aureus* to observe its effect on the pathogen. Through SEM analysis, it was observed that the coumarins-enriched extract causes bacterial inhibition by disrupting their cell wall, causing cytoplasm leakage. Most cells were found shrunk due to the loss of cytoplasm or necrosis, indicating cell death. This also proves the enriched extract isolated from *Aegle marmelos* will be effective against diabetic foot ulcers (Suaya et al., 2013).

The coumarins-enriched extract isolated from the *Aegle marmelos* was found to contain approximately 50% of the total coumarins content as a sum total of marmelosin, marmesin, psoralen and scopoletin. Marmelosin was found to be the predominant compound in the fraction with more than 30% w/w content. Following this, around 9% w/w marmesin, 4% w/w psoralen, and 2% scopoletin was also quantified. A trace amount of aegeline and umbelliferone were also found, whose content was less than 0.70% and 2.0% w/w of the total extract. The content of the coumarins was quantified by three different high-throughput methods, viz., HPTLC, HPLC, and ^1^H NMR.

The focus of the present study is the development of the Indian Pharmacopoeia Phytopharmaceutical Ingredient-Reference Standard (IP-PPI-RS) of *Aegle marmelos* by characterizing and quantifying the six major and valuable bioactive markers with known therapeutic potential. A novel method with simultaneous analysis of all the coumarins was developed which will be highly valuable for researchers and pharmaceutical industries. Indian Pharmacopoeia Commission (IPC) has taken the lead in developing the phytopharmaceutical ingredients reference standards, which may be competing with other pharmaceutical drugs in upcoming years. Indian Pharmacopoeia is giving long-standing recognition to plant-based drugs by developing and validating the PPI monographs and confirming their suitability and permeability for every phytopharmaceutical industry.

## 5 Conclusion

The present study entails a simple, sophisticated and inexpensive method of six major bioactive compound(s) enrichment from *Aegle marmelos* fruit. A targeted approach was taken for bioactive markers selection and their quantification in the enriched extract. The study is supported by implying multiple techniques for the detection and validation of the compound(s) content, making it more reliable. Simultaneous quantification of the targeted coumarins by HPTLC and by other techniques, viz., - HPLC and ^1^H NMR, was found to be precise, adding a new dimension in compound(s) characterization in plant extracts. On the basis of *in silico* and *in vitro* assays, enriched extract of *Aegle marmelos* fruit was found to be a potent anti-diabetic and anti-inflammatory agent, with marmelosin and marmesin showing the best activities against the enzymes targeted for the above-mentioned assays. This study is a major milestone which acts as a scaffold for the phytopharmaceutical drug. The depicted coumarins-enriched extract is an intermediate product before the phytopharmaceutical drug(s) formulation.

## Declaration of Competing Interest

The authors declare that they have no known competing financial interests or personal relationships that could have appeared to influence the work reported in this paper.
