# Supplementary material for: Comprehensive chemo-profiling of coumarins enriched extract derived from Aegle marmelos (L.) Correa fruit pulp, as an anti-diabetic and anti-inflammatory agent
Source: Saudi Pharm J. 2023 Jul 25;31(9):101708. doi: 10.1016/j.jsps.2023.101708 (PMC10410585; doi:10.1016/j.jsps.2023.101708)
Supplement: Supplementary data 1 [file mmc1.docx]

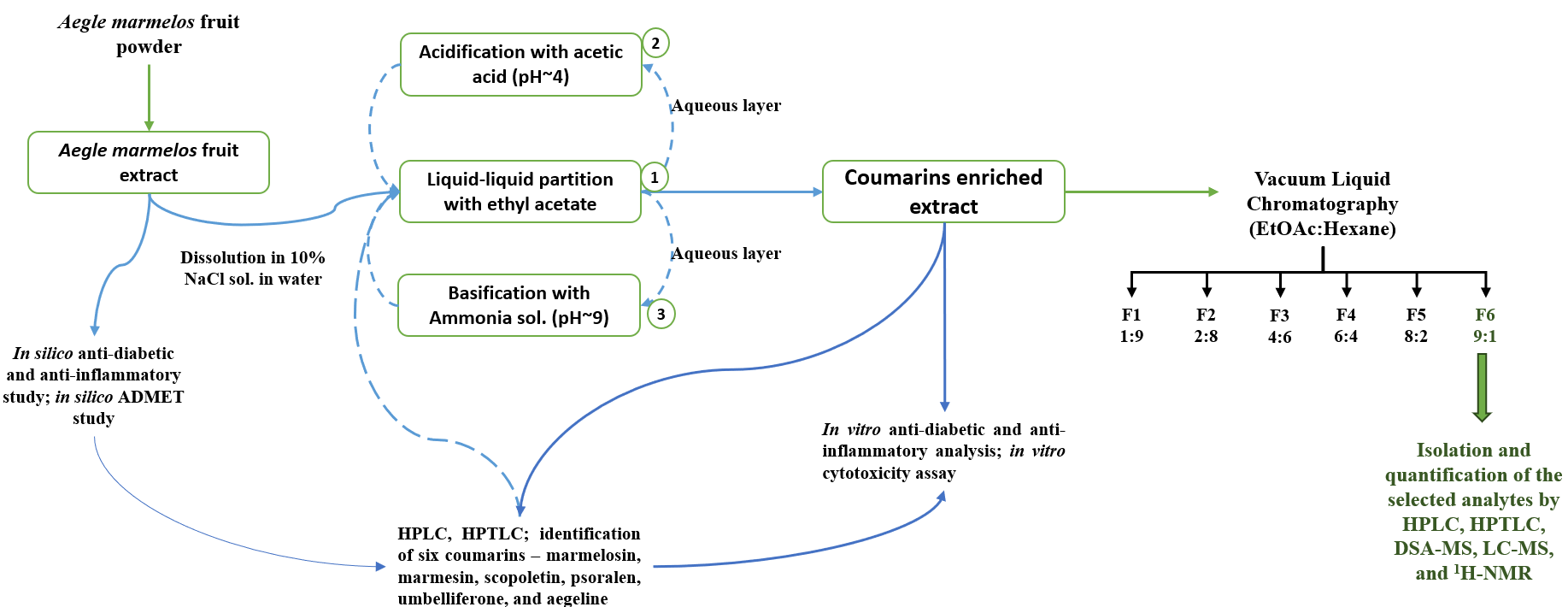


**Supplementary Figure 1:** Flow diagram of Coumarins enrichment process development from *Aegle marmelos* fruit pulp powder. Coumarins extraction was first done by liquid-liquid partitioning; divided into three steps – (1) extract partition with ethyl acetate; (2) acidification and then partition with ethyl acetate; (3) basification and then partition with ethyl acetate.


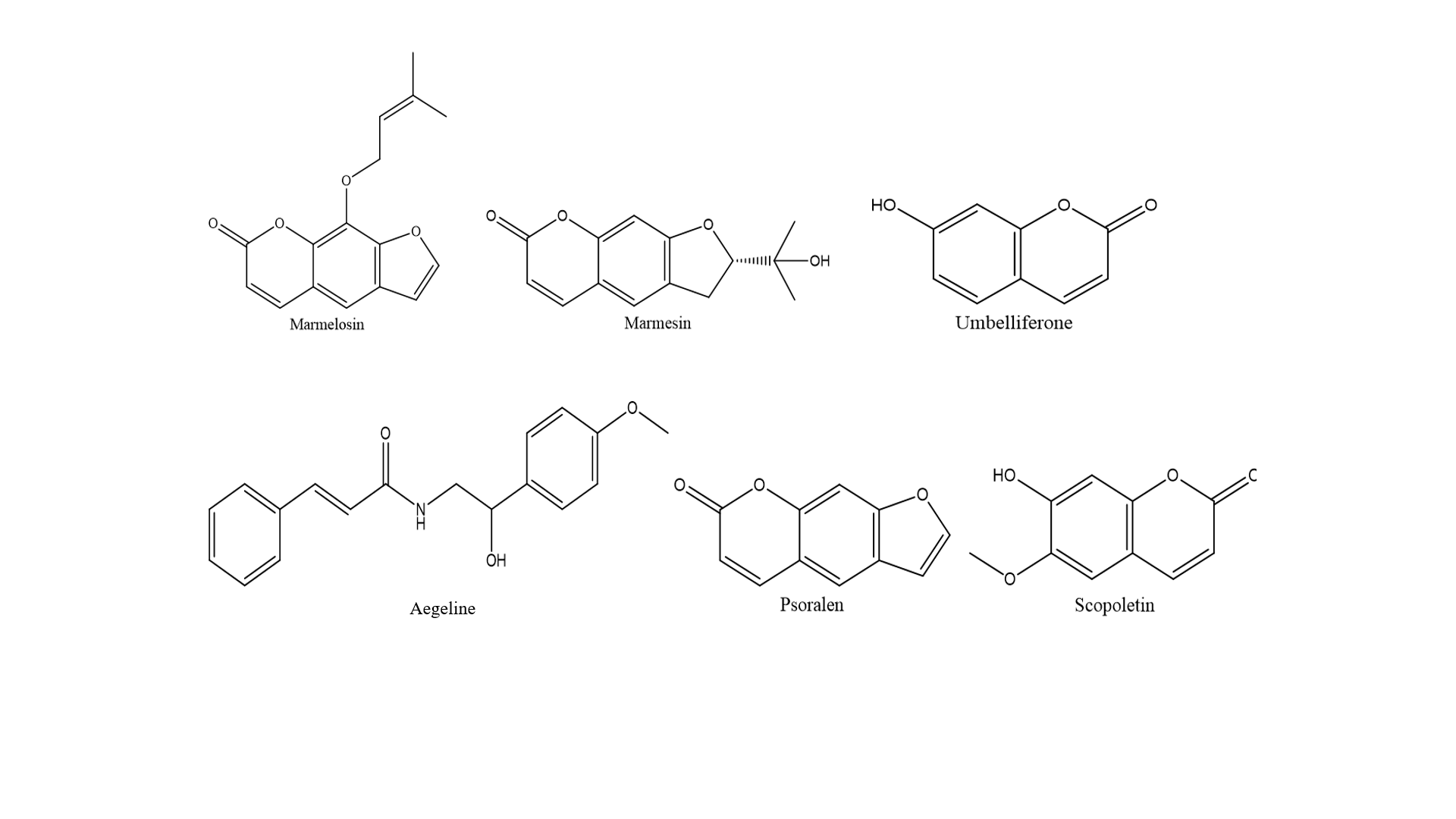


**Supplementary Figure 2:** Chemical structures of the targeted compounds enriched from *Aegle marmelos* fruit.


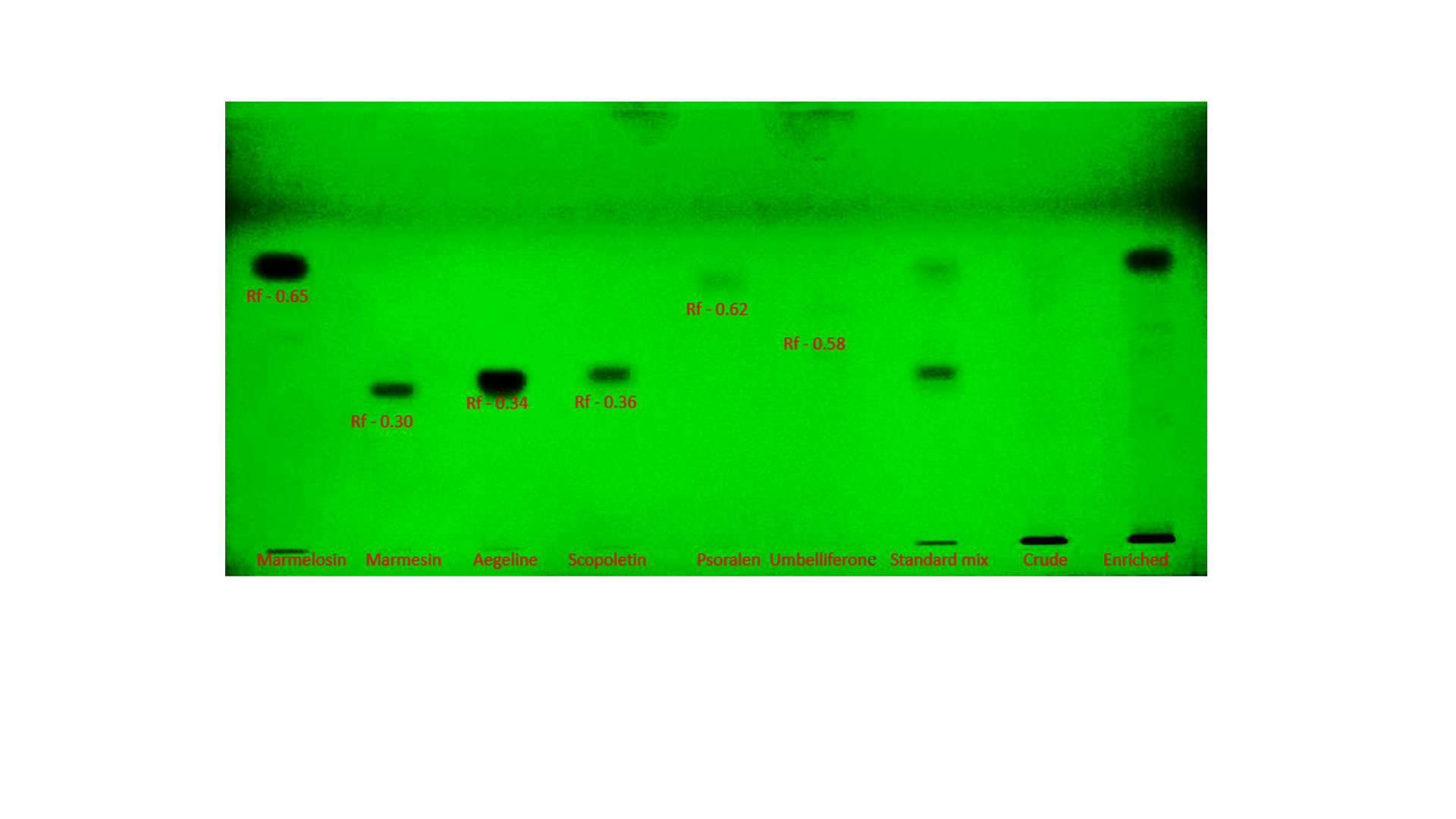


**A**

**
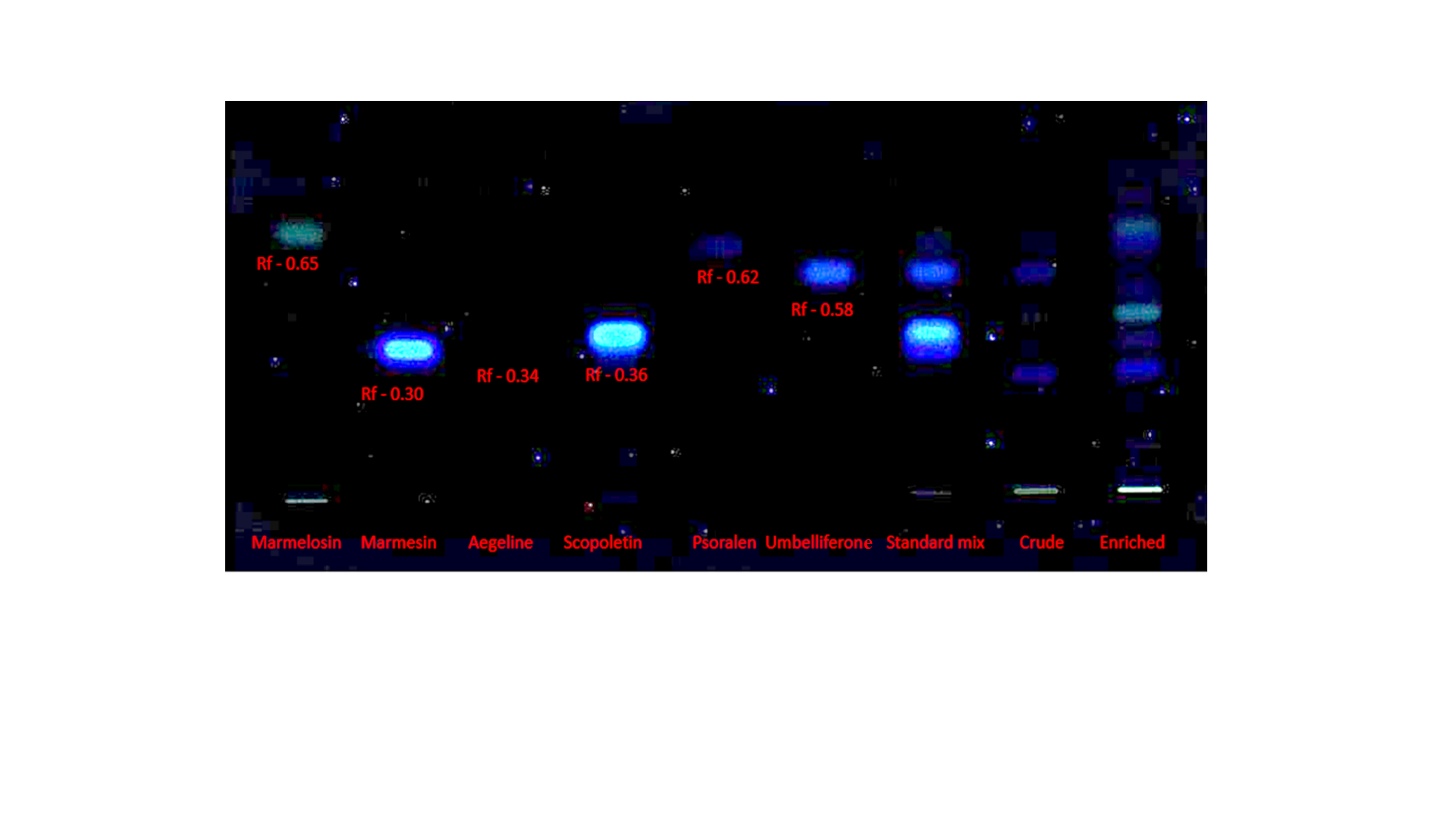
**

**B**

**Supplementary Figure 3:** HPTLC plate visualization at A) 254nm and B) 366nm. Track 1 – Marmelosin; Track 2 – Marmesin; Track 3 – Aegeline; Track 4 – Psoralen; Track 5 – Scopoletin; Track 6 – Umbelliferone; Track 7 – Standard mix; Track 8 – Crude extract; Track 9 – Enriched extract of *A. marmelos.*


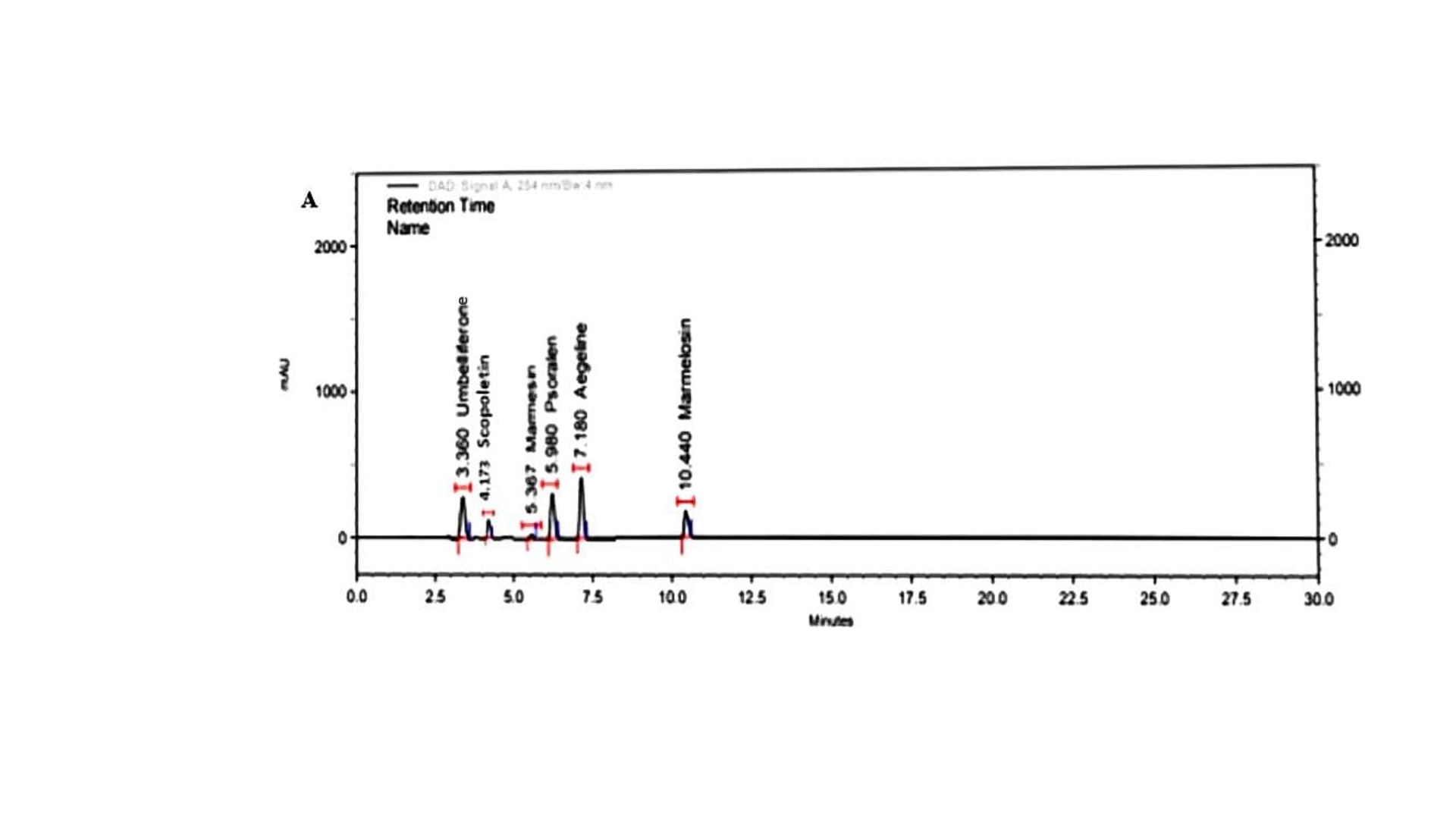

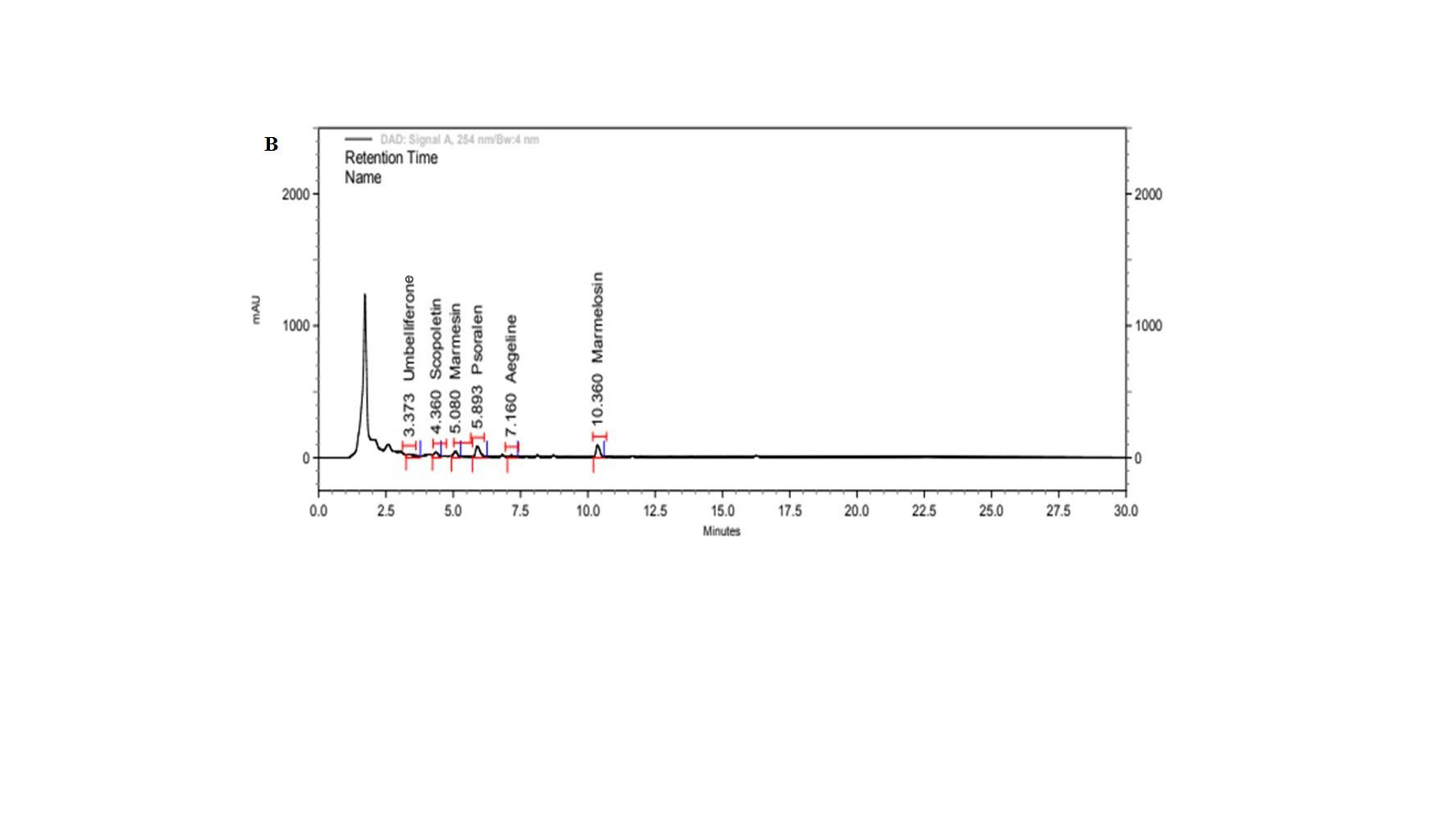

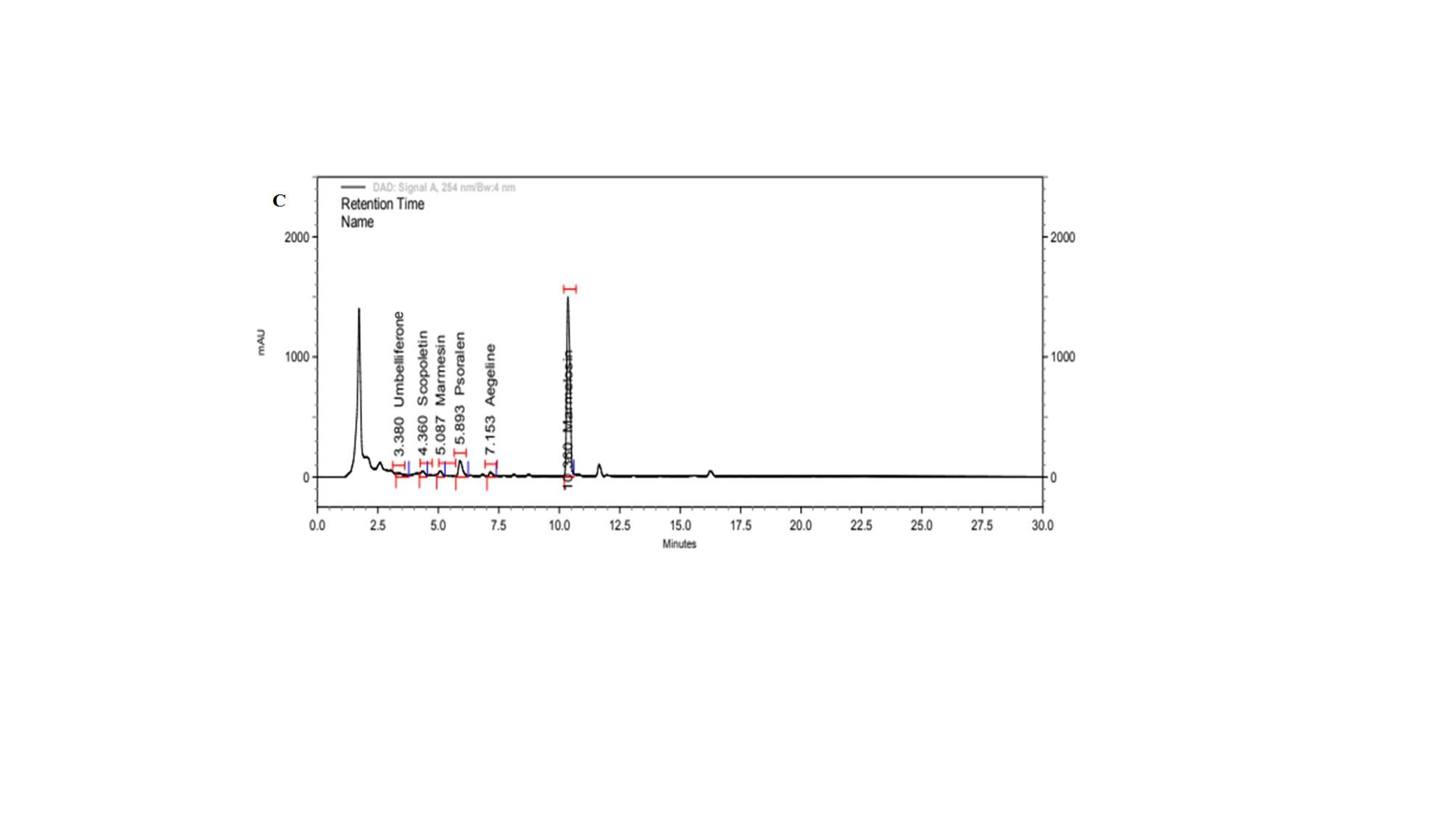


**Supplementary Figure 4:** HPLC analysis of *A. marmelos* crude extract and PPI with respect to the standards viz., *marmelosin*, *marmesin*, *aegeline*, *psoralen*, *scopoletin*, and *umbelliferone*. HPLC chromatogram A represents standard mix, B represents crude extract, and C represents PPI.


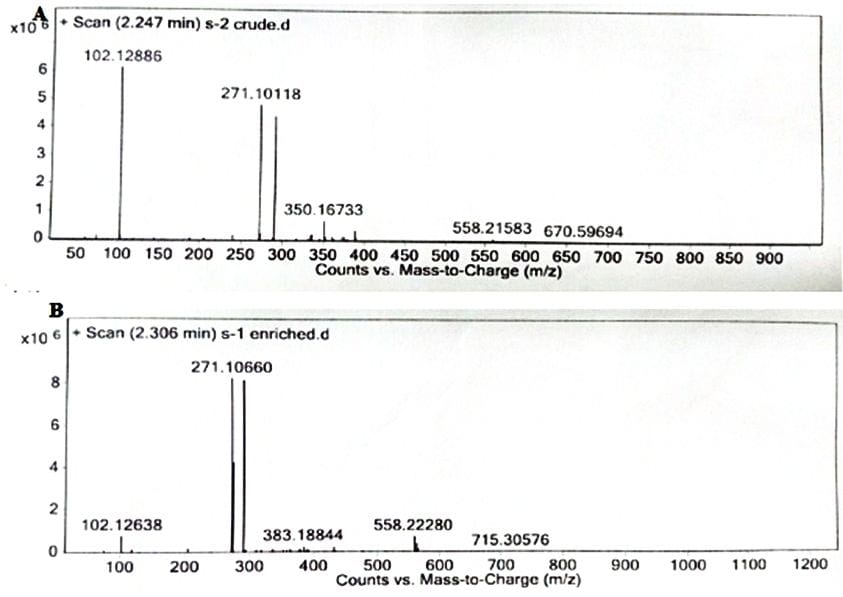


**Supplementary Figure 5:** LC-MS analysis of A) crude extract and B) enriched extract of *A. marmelos* fruit containing coumarins


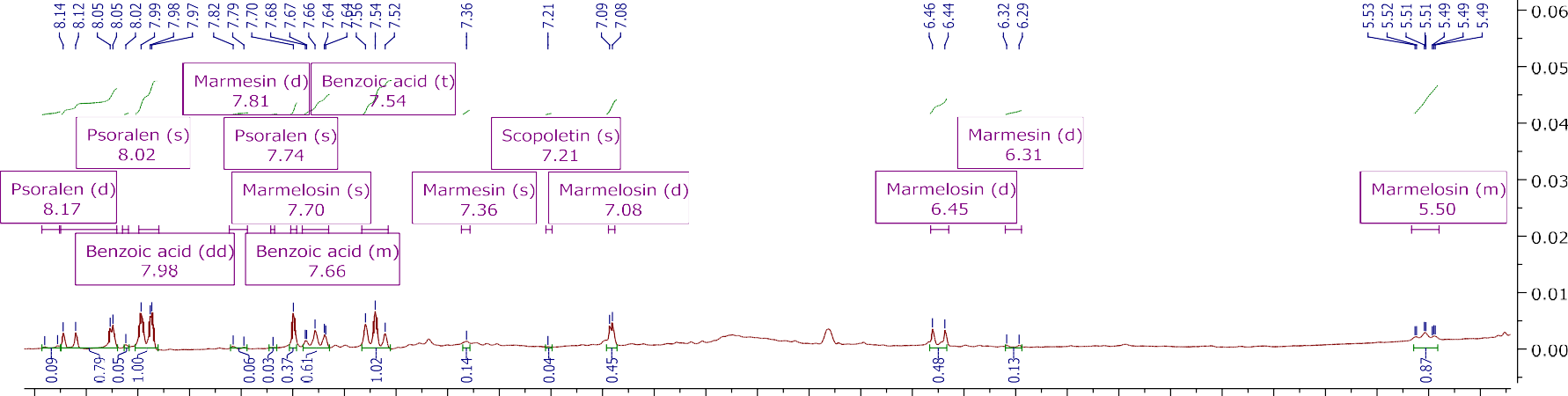

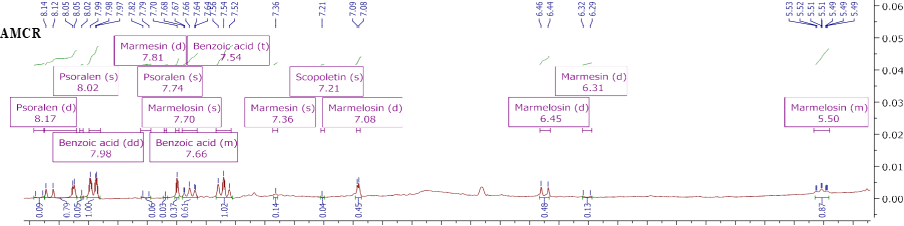


**Supplementary Figure 6a:** Stacked view of ^1^H-NMR spectrum of crude extract (represented as AMCR) and enriched extract (represented as AMPPI) of *A. marmelos* fruit


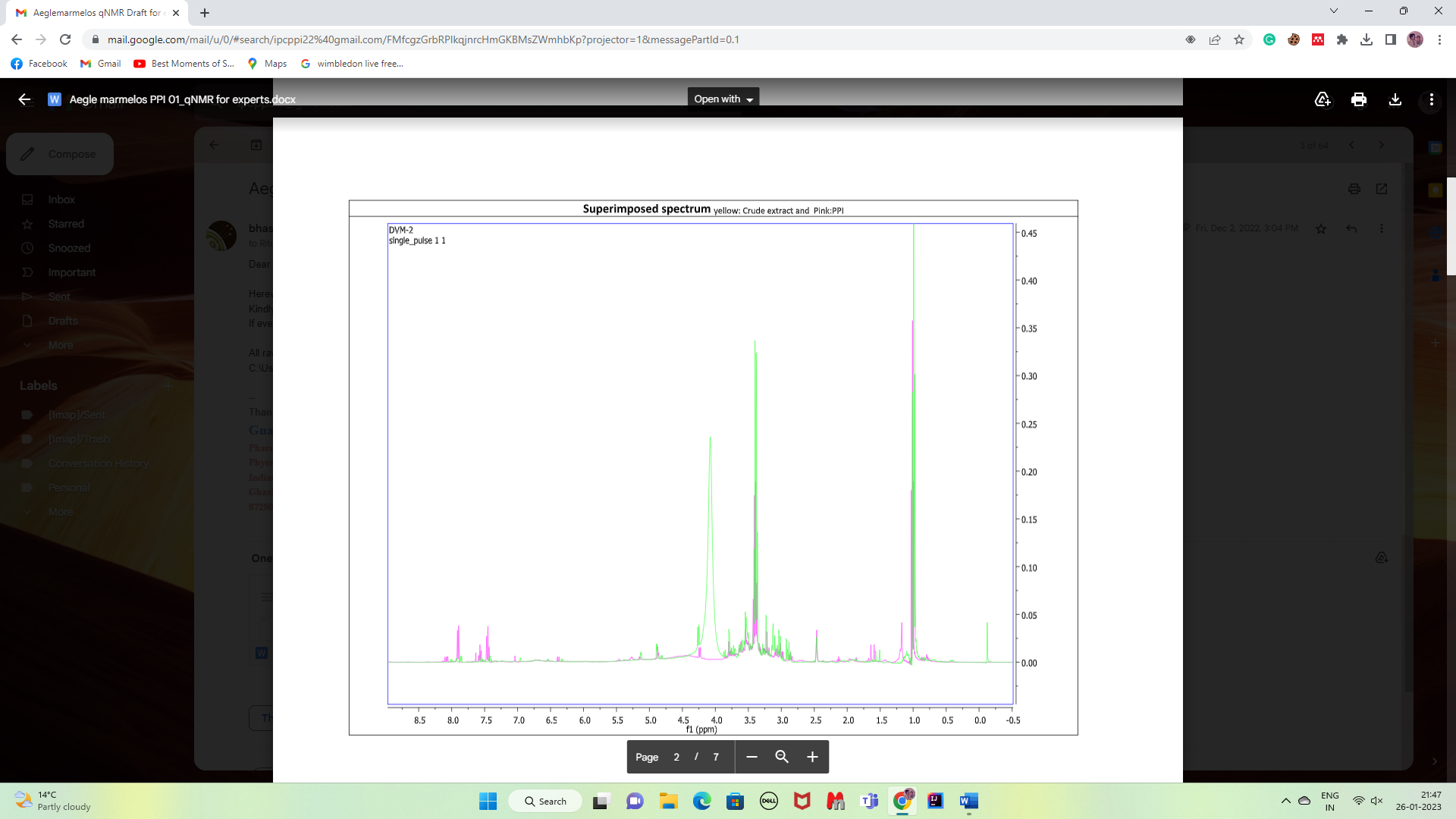


**Supplementary Figure 6b:** Superimposed view of ^1^H-NMR spectrum of crude extract (green) and enriched extract (pink) of *A. marmelos* fruit.
