# Supplementary material for: Comprehensive chemo-profiling of coumarins enriched extract derived from Aegle marmelos (L.) Correa fruit pulp, as an anti-diabetic and anti-inflammatory agent
Source: Saudi Pharm J. 2023 Jul 25;31(9):101708. doi: 10.1016/j.jsps.2023.101708 (PMC10410585; doi:10.1016/j.jsps.2023.101708)
Supplement: Supplementary data 2 [file mmc2.docx]

**Supplementary Tables**

**Supplementary Table 1:** Gradient program for HPLC and LC-MS analysis.

| **Time** | **Parameter** | **Mobile phase A** | **Mobile phase B** |
| --- | --- | --- | --- |
| 0 | Mobile Phase | 50 | 50 |
| 2 | Flow rate | 0.8ml/min | 0.8ml/min |
| 5 | Mobile Phase | 35 | 65 |
| 7 | Flow rate | 1.0ml/min | 1.0ml/min |
| 12 | Mobile Phase | 20 | 80 |
| 20 | Mobile Phase | 10 | 90 |
| 25 | Mobile Phase | 25 | 75 |
| 30 | Mobile Phase | 50 | 50 |

**Supplementary Table 2:** Physicochemical profiling of *Aegle marmelos* fruit powder

| **Parameters** | **Result** | **Limits given in USP** |
| --- | --- | --- |
| Loss on Drying | 5.78 ± 0.76 % w/w | NMT 6.0% w/w |
| Water soluble extractive | 69.23 ± 0.18 % w/w | NLT 10.0% w/w |
| Alcohol soluble extractive | 45.86 ± 0.49 % w/w | NLT 40.0% w/w |
| Total Ash | 2.27 ± 0.02 % w/w | NMT 4.0% w/w |
| Acid Insoluble Ash | 0.87 ± 0.01 % w/w | NMT 1.0% w/w |
| Heavy metals | 6.2 ± 0.5 ppm | NMT 30ppm |

NMT: Not more than; NLT: Not less than; w/w: weight/weight; ppm: parts per million; Values are represented as Mean±SD.

**Supplementary Table 3:** Pharmacological assay results of *Aegle marmelos* fruit crude and enriched extract

| **Pharmacological Tests** | **Crude extract** | **PPI** | **Standard drug** |
| --- | --- | --- | --- |
| **Anti-oxidant capacity** | 1100.62 ± 86.82 AAE | 1918.05 ± 153.20 AAE | Not Applicable |
| **EC50 as anti-oxidant agent** | 996.23 ± 2.88 µg | 628.15 ± 0.96 µg | 413.59 ± 4.03 µg |
| **IC_50_ value for anti-hyperglycemic activity** | 68.35 ± 2.48 µg | 23.85 ± 0.78 µg | 27.23 ± 0.84 µg (Metformin) |
| **IC_50_ value for anti-proteinase activity** | 60.79 ± 1.39 µg | 38.21 ± 0.44 µg | 35.77 ± 0.38 µg (Ibuprofen) |

AAE: Ascorbic Acid Equivalent; Values are represented as Mean±SD

**Supplementary Table 4:** Molecular interaction data of all molecules for ACE (4CA5), Beta protein (12RR4H), 1AZM (Diuretics), and LOX-2 protein

| **α-amylase (2QV4)** | | | | | | |
| --- | --- | --- | --- | --- | --- | --- |
| **Molecule** | **Pk** | **VINA score (kcal/mol)** | **Pocket volume** | **Binding residues** | **Coordinates** | **Binding size Coordinates** |
| Acarbose | C1 | -8.1 | 496 | **Chain A**: trp58 trp59 tyr62 gln63 his101 gly104 asn105 ala106 val107 asp147 arg161 leu162 thr163 gly164 leu165 arg195 asp197 ala198 glu233 ile235 asn298 his299 asp300 his305 gly306 | 16, 51, 23 | 27, 27, 27 |
| Marmelosin | C1 | -7.4 | 496 | **Chain A**: trp58 trp59 tyr62 gln63 leu162 thr163 leu165 asp197 ile235 his299 asp300 his305 | 16, 51, 23 | 20, 20, 20 |
| Marmesin | C1 | -8.2 | 496 | **Chain A**: trp58 trp59 tyr62 gln63 leu165 asp197 his299 asp300 | 16, 51, 23 | 20, 20, 20 |
| Aegeline | C1 | -7.9 | 496 | **Chain A**: trp58 trp59 tyr62 gln63 his101 tyr151 leu162 thr163 leu165 arg195 asp197 ala198 ser199 lys200 his201 glu233 val234 ile235 his299 asp300 his305 | 16, 51, 23 | 25, 25, 25 |
| Psoralen | C1 | -6.3 | 496 | **Chain A**: trp58 trp59 tyr62 gln63 leu162 thr163 leu165 asp197 glu233 his299 asp300 | 16, 51, 23 | 18, 18, 18 |
| Umbelliferone | C1 | -6.5 | 496 | **Chain A**: trp58 trp59 tyr62 gln63 his101 leu162 thr163 leu165 arg195 asp197 ala198 glu233 his299 asp300 | 16, 51, 23 | 18, 18, 18 |
| Scopoletin | C2 | -6.5 | 496 | **Chain A**: trp58 trp59 tyr62 gln63 his101 leu162 leu165 arg195 asp197 ala198 glu233 ile235 his299 asp300 | 16, 51, 23 | 17, 17, 17 |
| **β-glucosidase (2ZOX)** | | | | | | |
| Miglitol | C2 | -5.4 | 481 | **Chain A**: gln17 his120 phe121 asn164 gln165 val168 met172 phe179 phe225 tyr309 trp345 glu373 trp417 glu424 trp425 phe433 | -27, 2, 8 | 18, 18, 18 |
| Marmelosin | C1 | -8.6 | 1020 | **Chain A**: asn167 val168 val171 met172 val227 leu229 ala246 phe249 his250 arg312 ile314 ile326 leu327 ala330 ile332 phe334 | -29, 11, -2 | 20, 20, 20 |
| Marmesin | C1 | -8.7 | 1020 | Chain A: phe225 val227 leu229 ala246 thr310 arg312 ile314 leu327 ala330 ile332 phe334 trp345 | -29, 11, -2 | 20, 20, 20 |
| Aegeline | C1 | -8.6 | 1020 | Chain A: asn167 ser170 val171 tyr191 ala194 val227 leu229 ala246 phe249 his250 leu253 phe254 thr310 arg312 ile314 ile326 leu327 ala330 ile332 phe334 trp345 | -29, 11, -2 | 25, 25, 25 |
| Psoralen | C1 | -7.7 | 1020 | **Chain A**: val227 leu229 ala246 phe249 his250 arg312 ile314 ile326 leu327 ala330 ile332 phe334 | -29, 11, -2 | 18, 18, 18 |
| Scopoletin | C1 | -6.5 | 1020 | **Chain A**: val227 leu229 ala246 his250 thr310 arg312 ile314 leu327 ile332 phe334 trp345 | -29, 11, -2 | 18, 18, 18 |
| Umbelliferone | C1 | -6.5 | 1020 | Chain A: asp113 val114 val117 thr118 phe193 thr195 tyr199 ala200 ala202 ser203 ser204 ser207 phe289 phe290 asn293 tyr308 | -29, 11, -2 | 17, 17, 17 |
| **Pancreatic lipase (2OXE)** | | | | | | |
| Sibutramine | C2 | -5.7 | 505 | **Chain A**: phe96 tyr133 pro199 ser231 gly233 phe234 trp271 gly273 gly276 phe277 | 15, 0, 35 | 19, 19, 19 |
| Marmelosin | C4 | -7.4 | 417 | **Chain B**: tyr22 gly23 gln24 leu25 pro49 glu50 asp53 thr54 phe56 ile70 thr71 gly72 thr73 glu146 phe149 | -25, 23, 10 | 20,20,20 |
| Marmesin | C4 | -6.9 | 417 | **Chain B**: tyr22 gly23 gln24 leu25 pro49 glu50 phe56 ile70 thr71 gly72 thr73 glu146 phe149 leu150 | -25, 23, 10 | 20,20,20 |
| Aegeline | C4 | -7.2 | 417 | **Chain B**: tyr22 gly23 gln24 leu25 pro49 glu50 asp53 thr54 arg55 phe56 ile70 thr71 gly72 thr73 glu146 phe149 leu150 | -25, 23, 10 | 25, 25, 25 |
| Psoralen | C4 | -5.9 | 417 | **Chain B**: tyr22 gly23 gln24 leu25 thr54 phe56 thr71 gly72 thr73 glu146 phe149 | -25, 23, 10 | 18, 18, 18 |
| Scopoletin | C4 | -6.0 | 417 | **Chain B**: tyr22 gly23 gln24 leu25 thr54 phe56 thr71 gly72 thr73 glu146 phe149 leu150 | -25, 23, 10 | 18, 18, 18 |
| Umbelliferone | C4 | -6.7 | 417 | **Chain B**: tyr22 gly23 gln24 leu25 phe56 thr71 gly72 thr73 glu146 phe149 | -25, 23, 10 | 17, 17, 17 |
| **LOX-2 Protein** | | | | | | |
| Ibuprofene | C1 | -7.4 | 465 | Chain A: cys106 tyr107 gln108 asn173 ile174 lys175 thr385 leu389 his394 phe399 ile403 thr406 arg407 tyr408 thr409 leu410 his411 his627 | -13, -63, -18 | 35, 19, 35 |
| Marmelosin | C1 | -8.4 | 465 | Chain A: cys106 tyr107 gln108 arg145 tyr149 asn173 ile174 thr385 leu389 his394 phe399 ile403 thr406 arg407 tyr408 thr409 leu410 asp625 his627 | -13, -63, -18 | 35, 20, 35 |
| Umbelliferone | C1 | -7.1 | 465 | Chain A: asn173 ile174 lys175 thr385 leu389 ile403 thr406 arg407 tyr408 thr409 leu410 his411 asp625 | -13, -63, -18 | 35, 24, 35 |
| Scopoletin | C1 | -7.4 | 465 | Chain A: tyr107 asn173 ile174 lys175 thr385 leu389 ile403 thr406 arg407 tyr408 thr409 leu410 his411 | -13, -63, -18 | 35, 24, 35 |
| Psoralen | C1 | -8.1 | 465 | Chain A: tyr107 asn173 ile174 thr385 leu389 ile403 thr406 arg407 tyr408 thr409 leu410 his411 asp625 | -12, -63, -18 | 35, 24, 35 |
| Aegeline | C1 | -9.0 | 465 | Chain A: phe184 phe365 glu369 his373 leu374 his378 ile412 asn413 leu415 ala416 leu419 leu420 val427 phe438 gln560 leu607 leu610 ile676 | -12, -63, -18 | 35, 24, 35 |
| Marmesin | C1 | -8.4 | 465 | Chain A: gly11 glu12 ala13 phe88 arg90 trp109 leu172 asn173 ile174 lys175 tyr176 ser177 arg407 tyr408 arg618 pro624 asp625 | 16, -59, -25 | 35, 24, 35 |

**Supplementary Table 5:** ADMET study of all target analytes enriched from *Aegle marmelos* fruit and leaves.

| **Molecules** | **Lipinski violations** | **Water solubility (log mol/L)** | **OSIRIS Drug score** | **GPCR Ligand** | **P-Glycoprotein I and II Substrate** | **P-Glycoprotein I and II inhibitor** | **Skin permeability (log Kp)** | **Ion channel modulator** | **Kinase inhibitor** | **Nuclear receptor ligand** | **Protease Inhibitor** | **Intestinal absorption** |
| --- | --- | --- | --- | --- | --- | --- | --- | --- | --- | --- | --- | --- |
| **Marmel** | 0 | -3.08 | 0.18 | -0.37 | No | No | -2.351 | -0.02 | -0.56 | -0.18 | -0.5 | 97 |
| **Scopol** | 0 | -2.504 | 0.39 | -1.22 | No | No | -2.94 | -0.72 | -1.3 | -0.92 | -1.3 | 95 |
| **Umbell** | 0 | -2.131 | 0.29 | -1 | No | No | -2.6 | -0.65 | 0.95 | -0.81 | -1.16 | 94 |
| **Aegelin** | 0 | -3.313 | 0.83 | 0.19 | Yes | No | -2.97 | -0.22 | -0.23 | -0.04 | -0.05 | 93 |
| **Psoral** | 0 | -2.47 | 0.29 | -0.89 | Yes | No | -2.21 | -0.38 | -1.1 | -1.13 | -1.19 | 96 |
| **Marm** | 0 | -3.13 | 0.29 | -0.42 | No | No | -3.12 | -0.57 | -0.82 | -0.2 | -0.61 | 97 |
| **Molecules** | **Caco2 permeability** | **VDss log L/Kg** | **Fraction Unbound (Fu)** | **CYP 3a4 substrate** | **CYP 3a4/2c9 inhibitor** | **Renal OCT2 substrate** | **Total clearance log ml/min/kg** | **Skin Sensitization** | **Mutagenecity (AMES test)** | **Hepatotoxic** | **Maximum tolerant Dose log mg/kg/day** | **Maximum recommended daily dose (Human) (mg/kg bw/day)** |
| **Marmel** | 1.383 | 0.147 | 0.187 | Yes | No | No | 0.994 | No | No | No | 0.593 | 21.5 |
| **Scopol** | 1.18 | 0.034 | 0.363 | No | No | No | 0.73 | No | No | No | 0.614 | 17.4 |
| **Umbell** | 1.2 | 0.032 | 0.432 | No | No | No | 0.706 | No | No | Yes | 0.689 | 13.6 |
| **Aegelin** | 1.2 | 0.205 | 0.043 | Yes | No | No | 0.353 | No | Yes | No | -0.084 | 2.68 |
| **Psorale** | 0.29 | -0.13 | 0.301 | No | No | No | 0.773 | No | No | No | -0.253 | 1.6 |
| **Marm** | 1.15 | 0.507 | 0.367 | No | No | No | 0.647 | No | No | No | 0.272 | 1.61 |

**Supplementary Table 6:** HPTLC assay of all standards in crude and enriched extract of *Aegle marmelos* fruit

| **Samples** | **Compounds** | **Sample Area** | **Mean Standard Area** | **Sample amount (µg)** | **Mean Standard amount (µg)** | **Standard Purity** | **% Content** |
| --- | --- | --- | --- | --- | --- | --- | --- |
| **Crude** | **Marmelosin** | 2286.52 | 498.9 | 1.79 | 0.1123 | 99.5 | 2.89 |
| **PPI** |  | 27853.6 | 658.3 | 1.68 | 0.1123 | 99.5 | 28.43 |
| **Crude** | **Marmesin** | 853.4 | 236.2 | 1.79 | 0.1188 | 99.5 | 2.41 |
| **PPI** |  | 2761.4 | 359.8 | 1.68 | 0.1188 | 99.5 | 5.45 |
| **Crude** | **Psoralen** | 286.4 | 221 | 1.79 | 0.2801 | 99.5 | 2.04 |
| **PPI** |  | 271.6 | 118.6 | 1.68 | 0.2801 | 99.5 | 3.84 |
| **Crude** | **Scopoletin** | 99.6 | 2284.3 | 1.79 | 5.0013 | 99.5 | 1.22 |
| **PPI** |  | 339 | 3186.4 | 1.68 | 5.0013 | 99.5 | 3.18 |
| **Crude** | **Umbelliferone** | 211 | 956 | 1.79 | 1.0187 | 99.5 | 1.26 |
| **PPI** |  | 325 | 1028.3 | 1.68 | 1.0187 | 99.5 | 1.93 |
| **Crude** | **Aegeline** | 118 | 1286 | 1.79 | 0.4838 | 99.5 | 0.25 |
| **PPI** |  | 502.6 | 2215.3 | 1.68 | 0.4838 | 99.5 | 0.66 |

**Supplementary Table 7:** HPLC assay of all six standards in crude extract and PPI of *Aegle marmelos* fruit

| Samples | Compounds | Sample Area | Standard Area | Sample weight | Standard weight (mg) | Standard dilution (ml) | Standard Purity | % Content |
| --- | --- | --- | --- | --- | --- | --- | --- | --- |
| Crude | Marmelosin | 2845094 | 3159734 | 1.79 | 0.1123 | 5 | 99.5 | 2.84 |
| PPI |  | 29347110 | 3159734 | 1.68 | 0.1123 | 5 | 99.5 | 31.20 |
| Crude | Marmesin | 610023 | 515109 | 1.79 | 0.1188 | 5 | 99.5 | 3.95 |
| PPI |  | 1288026 | 515109 | 1.68 | 0.1188 | 5 | 99.5 | 8.89 |
| Crude | Psoralen | 1653394 | 5529506 | 1.79 | 0.2801 | 5 | 99.5 | 2.35 |
| PPI |  | 2678944 | 5529506 | 1.68 | 0.2801 | 5 | 99.5 | 4.06 |
| Crude | Scopoletin | 1098772 | 40682455 | 1.79 | 5.0013 | 2 | 99.5 | 1.52 |
| PPI |  | 1379703 | 40682455 | 1.68 | 5.0013 | 2 | 99.5 | 2.03 |
| Crude | Umbelliferone | 1394050 | 5525093 | 1.79 | 1.0187 | 1 | 99.5 | 1.44 |
| PPI |  | 1553648 | 5525093 | 1.68 | 1.0187 | 1 | 99.5 | 1.71 |
| Crude | Aegeline | 64304 | 6742426 | 1.79 | 0.4838 | 5 | 99.5 | 0.13 |
| PPI |  | 334358 | 6742426 | 1.68 | 0.4838 | 5 | 99.5 | 0.72 |

**Supplementary Table 8:** DSA-MS analysis of crude extract and PPI of *Aegle marmelos* fruit

| **Compound(s)** | **Exact mass** | **Obtained mass in enriched** | **Obtained mass in Crude extract** | **Fragmentation pattern** |
| --- | --- | --- | --- | --- |
| **Marmelosin** | 270.28 | 280.2334 | 280.2358 | M+Na^+^ |
|  |  | 312.2796 | 334.3005 | 2M+Na^+^+ACN |
|  |  | 334.2944 | 335.3079 | 2M+Na^+^+ACN+H |
| **Marmesin** | 246.2 | 180.1526 | 180.1543 | M/2+Na^+^ |
|  |  | 182.1722 | 181.1509 | M/2+Na^+^+H^+^ |
| **Aegeline** | 297.3 | 306.8331 | 307.2489 | M+Na^+^+H^+^ |
|  |  | 307.8489 | 308.2582 | M+Na^+^+2H^+^ |
| **Psoralen** | 186.16 | 335.295 | 335.295 | 2M+Na^+^+2H^+^ |
| **Scopoletin** | 192.17 | 198.1689 | 198.1699 | M+6H^+^ |
|  |  | 200.1849 | 200.1808 | M+8H^+^ |
| **Umbelliferone** | 162.14 | 855.98785 | - | 5M+2Na^+^ |
|  |  | 872.97083 | 873.05446 | 5M+K^+^+Na^+^ |

**Supplementary Table 9:** LC-MS analysis of crude and enriched extract of *A. marmelos* fruit

| **Compound(s)** | **Exact mass** | **Obtained mass in PPI** | **Obtained mass in Crude extract** | **Fragmentation pattern** |
| --- | --- | --- | --- | --- |
| **Marmelosin** | 270.28 | 271.1066 | 271.1066 | M+H^+^ |
|  |  | 272.10196 | 272.10196 | M+2H^+^ |
|  |  | 102.12638 | 102.12638 | M/3+H^+^ |
| **Marmesin** | 246.2 | 288.13124 | 288.13124 | M+ACN |
|  |  | 289.12551 | 289.12551 | M+ACN+H^+^ |
| **Aegeline** | 297.3 | - | 350.16733 | M+2Na^+^+7H^+^ |
| **Psoralen** | 186.16 | 715.30576 |  | 3M+2ACN+Na^+^+K^+^ |
|  |  |  | 670.59694 | 3M+2ACN+Na^+^+9H^+^ |
| **Scopoletin** | 192.17 | 383.18844 | - | 2M^+^ |
|  |  | - | 388.40061 | 2M+4H^+^ |
| **Umbelliferone** | 162.14 | 855.98785 | - | 5M+2Na^+^ |
|  |  | 872.97083 | 873.05446 | 5M+K^+^+Na^+^ |

**Supplementary Table 10:** qNMR assay of all six standards in crude extract and enriched extract of *Aegle marmelos* fruit

| **Sample** | **Compounds** | **(nIC)** | **(nt)** | **(Intt)** | **(IntIC)** | **(MWt)** | **(MWIC)** | **(mIC)** | **(mt)** | **(PIC)** | **% Purity of Compound** |
| --- | --- | --- | --- | --- | --- | --- | --- | --- | --- | --- | --- |
| **Crude** | **Marmelosin** | 1 | 1 | 0.1 | 0.54 | 270.28 | 122.12 | 2 | 38.2 | 99 | 2.124395 |
| **PPI** |  | 1 | 1 | 0.112 | 0.51 | 270.28 | 122.12 | 6 | 9.87 | 99 | 29.25124 |
| **Crude** | **Marmesin** | 1 | 1 | 0.11 | 0.54 | 246.26 | 122.12 | 2 | 38.2 | 99 | 2.336834 |
| **PPI** |  | 1 | 1 | 0.023 | 0.51 | 246.26 | 122.12 | 6 | 9.87 | 99 | 6.006951 |
| **Crude** | **Psoralen** | 1 | 1 | 0.057 | 0.54 | 186.16 | 122.12 | 2 | 38.2 | 99 | 1.210905 |
| **PPI** |  | 1 | 1 | 0.017 | 0.51 | 186.16 | 122.12 | 6 | 9.87 | 99 | 4.43992 |
| **Crude** | **Scopoletin** | 1 | 1 | 0.04 | 0.54 | 192.16 | 122.12 | 2 | 38.2 | 99 | 0.849758 |
| **PPI** |  | 1 | 1 | 0.01 | 0.51 | 192.16 | 122.12 | 6 | 9.87 | 99 | 2.611718 |
| **Crude** | **Aegeline** | 1 | 1 | 0.0035 | 0.54 | 297.30 | 122.12 | 2 | 38.2 | 99 | 0.074354 |
| **PPI** |  | 1 | 1 | 0.001 | 0.51 | 297.30 | 122.12 | 6 | 9.87 | 99 | 0.261172 |
| **Crude** | **Umbelliferone** | 1 | 1 | 0.042 | 0.54 | 162.14 | 122.12 | 2 | 38.2 | 99 | 0.892246 |
| **PPI** |  | 1 | 1 | 0.0043 | 0.51 | 162.14 | 122.12 | 6 | 9.87 | 99 | 1.123039 |
